# Dose-Response of Aerobic Exercise on Cognition: A Community-Based, Pilot Randomized Controlled Trial

**DOI:** 10.1371/journal.pone.0131647

**Published:** 2015-07-09

**Authors:** Eric D. Vidoni, David K. Johnson, Jill K. Morris, Angela Van Sciver, Colby S. Greer, Sandra A. Billinger, Joseph E. Donnelly, Jeffrey M. Burns

**Affiliations:** 1 University of Kansas Alzheimer’s Disease Center, University of Kansas Medical Center, Fairway, Kansas, United States of America; 2 Department of Psychology, University of Kansas, Lawrence, Kansas, United States of America; 3 Department of Physical Therapy and Rehabilitation Science, University of Kansas Medical Center, Kansas City, Kansas, United States of America; 4 Department of Internal Medicine, University of Kansas Medical Center, Kansas City, Kansas, United States of America; Kurume University School of Medicine, JAPAN

## Abstract

Epidemiological studies suggest a dose-response relationship exists between physical activity and cognitive outcomes. However, no direct data from randomized trials exists to support these indirect observations. The purpose of this study was to explore the possible relationship of aerobic exercise dose on cognition. Underactive or sedentary participants without cognitive impairment were randomized to one of four groups: no-change control, 75, 150, and 225 minutes per week of moderate-intensity semi-supervised aerobic exercise for 26-weeks in a community setting. Cognitive outcomes were latent residual scores derived from a battery of 16 cognitive tests: Verbal Memory, Visuospatial Processing, Simple Attention, Set Maintenance and Shifting, and Reasoning. Other outcome measures were cardiorespiratory fitness (peak oxygen consumption) and measures of function functional health. In intent-to-treat (ITT) analyses (n = 101), cardiorespiratory fitness increased and perceived disability decreased in a dose-dependent manner across the 4 groups. No other exercise-related effects were observed in ITT analyses. Analyses restricted to individuals who exercised per-protocol (n = 77) demonstrated that Simple Attention improved equivalently across all exercise groups compared to controls and a dose-response relationship was present for Visuospatial Processing. A clear dose-response relationship exists between exercise and cardiorespiratory fitness. Cognitive benefits were apparent at low doses with possible increased benefits in visuospatial function at higher doses but only in those who adhered to the exercise protocol. An individual’s cardiorespiratory fitness response was a better predictor of cognitive gains than exercise dose (i.e., duration) and thus maximizing an individual’s cardiorespiratory fitness may be an important therapeutic target for achieving cognitive benefits.

**Trial Registration:**

ClinicalTrials.gov NCT01129115

## Introduction

As the population grows older, public health must prioritize preventive strategies to reduce age-related functional and cognitive disability [1]. Aerobic exercise (AEx)–most commonly walking–is accessible, cost effective [2], has proven health benefits [3], and may protect against cognitive decline and dementia [4–7]. Unlike with prescription drugs, however, the minimum and maximum effective exercise doses remain unknown.

Consensus guidelines, based on indirect data from epidemiological and prospective studies [8] state a dose-response relationship exists between exercise and health benefits. Some exercise is better than none and higher doses generally convey greater benefit. Epidemiological studies suggest this dose-response relationship also applies to cognitive outcomes, with greater cognitive performance and lower dementia risk in individuals who have greater levels of physical activity [6, 7, 9]. Higher levels of cardiorespiratory fitness are associated with slower longitudinal cognitive decline [10]. And in a parallel literature, exercise has demonstrated some effect in cognitively impaired older adults [11–13]. However, no direct data from randomized trials exists to support these indirect observations and meta-analyses have not clearly demonstrated a linear relationship between fitness and cognition [14, 15].

This study examines the potential dose-response relationship of AEx on cognitive and functional outcomes for maximizing exercise-related cognitive benefit in older adults. To accomplish this, we performed a pilot randomized controlled 26-week trial of three doses of AEx representing 50%, 100%, or 150% of the recommended exercise dose of 150 minutes per week [16]. Our goals were to test the ability of a community-based, semi-supervised exercise program to deliver a rigorously controlled exercise dose and perform a preliminary test of our hypothesis that low doses of AEx would provide some cognitive and functional benefits and that benefits would increase at higher doses of exercise.

## Methods

### Study Design

The Trial of Exercise on Aging and Memory (TEAM: ClinicalTrials.gov, NCT01129115; trial active between 2/1/2010–2/18/2014) was a 26-week pilot study of AEx dose in individuals 65 years and older without cognitive impairment. Based on public health recommendations to attain at least 150 minutes per week (min/wk) of moderate intensity AEx [16, 17], we randomly assigned participants to 1 of 4 intervention arms: no change in current physical activity (control), 75 min/wk, 150 min/wk, or 225 min/wk. We modeled the intervention on a prior dose-response study of AEx [18], altering the dose through exercise duration while keeping exercise intensity consistent across groups. Cognition, cardiorespiratory fitness, and functional health were measured at baseline and post-intervention.

### Participants

Participants were recruited as a convenience sample of volunteers through print and online advertising, community talks, and existing databases of individuals willing to be in research studies. Interested individuals underwent a telephone screen of medical history for inclusion and exclusion criteria. Participants had to be at least 65, sedentary or underactive as defined by the Telephone Assessment of Physical Activity [19], and free of cognitive impairment. Participants could not be insulin-dependent, have significant hearing or vision problems, uncontrolled hypertension, or have had recent history (<2 years) of major cardiorespiratory, musculoskeletal or neuropsychiatric impairment.

After telephone screen, we scheduled candidate participants who remained interested for baseline evaluation visits. We obtained written, informed consent under a study protocol (#11883) approved by the University of Kansas Medical Center Institutional Review Board, which also acted as the human subjects research compliance entity for the YMCA of Greater Kansas City. The baseline evaluation included a thorough clinical examination by a trained clinician that included a Clinical Dementia Rating (CDR) to exclude the presence of dementia [20]. A trained psychometrician administered a comprehensive cognitive testing battery and the clinical and psychometric test results were reviewed and discussed at a weekly consensus conference that included clinicians, a neuropsychologist, and raters. All eligible participants were deemed cognitively normal as defined by a CDR of 0 (no dementia) and determination of no clinical significant impairment on cognitive tests as determined by clinical review. Eligible individuals then underwent physical function and cardiorespiratory fitness testing. The cognitive test battery at screening was used as baseline. The same testing was conducted again after 26 weeks of intervention.

### Randomization and Blinding

Participants were block randomized, stratified by age (split at 75) and sex, to ensure the groups were well-matched. Our enrollment goal of 100 was based on reported exercise-related effect sizes on cognition [21], with a goal of powering a definitive dose-response trial. Intervention allocation was performed after completion of baseline testing by staff not involved with outcome measure testing. One investigator constructed the allocation schedule using Random Allocation Software and stored it in an electronic, password protected file prior to study start [22]. Psychometric and exercise testers were blinded to the participant’s intervention arm at all times.

### Intervention

Participants were asked not to start or stop any new regular physical activities other than those prescribed by the study team. The control group was instructed not to change their previous sedentary or underactive level of physical activity.

For those randomized to an exercise group, the intervention was conducted at their nearest Young Men’s Christian Association of Greater Kansas City (YMCA) under the guidance of certified personal trainers who were trained and monitored by study staff. Personal trainers oversaw personalized prescription for weekly duration and intensity under the direction of the study team. At each session, participants manually recorded the duration of exercise on an exercise study log. All exercise groups began with a goal of 60 total minutes during Week 1 and increased their goal by approximately 21 min/wk until they achieved their exercise duration (i.e., 75, 150 or 225 min/wk, S2 Table). Participants exercised 3–5 days a week, never more than 50 minutes a day to reduce likelihood of overuse injury. Intensity was prescribed as a target heart rate zone (F4 or FT4, Polar Electro Inc., Lake Success, NY) based on percentage of heart rate reserve (HRR) as calculated by the Karvonen formula. In the first 4 weeks of exercise, the target heart rate zone was 40–55% of HRR. In Weeks 5–18, it was 50–65% of HRR. In weeks 19–26, it was 60–75% of HRR. We selected a 26-week intervention period to maximize physiological adaptation [23] without overburdening participants.

Trainers supervised all exercise sessions for the first 6 weeks of exercise after which direct supervision occurred during at least 1 session a week. Treadmill walking served as the primary exercise modality but participants were allowed to use a different aerobic modality (e.g. elliptical) once a week. Facility and trainer fees were paid by the study. No other compensation was provided.

### Adherence and Safety

Trainers asked about changes in health status (adverse events [AE]) and medication changes every visit. Study staff also inquired about AEs during phone calls every 6 weeks and during incidental contact at weekly YMCA visits. An independent safety committee reviewed AEs quarterly.

Intent-to-treat analyses were performed on all enrollees (n = 101, ITT cohort). Per-protocol analyses (PP, n = 77) excluded individuals not achieving 80% of their exercise duration goal, those who engaged in other activities (e.g. resistance training), or those who withdrew. All participants were asked about exercise outside the intervention at each phone call.

### Outcomes

Our primary physical function outcome was 26-week percent difference (%Δ) in cardiorespiratory fitness, measured as peak oxygen consumption normalized to body mass (VO_2_ peak, ml/kg/min) [24, 25]. VO_2_ peak was as the highest observed value during a maximal cardiopulmonary exercise test following the Cornell modified Bruce protocol [26]. To maximize validity of the test, all participants were required to achieve a respiratory exchange ratio of at least 1.0 before enrollment and exercise prescription.

Our primary objective measure of functional health was the Physical Performance Test (PPT) [27]. The Late-Life Function and Disability Index (LLFDI) and Short Form 36 Health Survey Physical and Mental Component Scores (SF-36v2, Quality Metric, Inc.) were considered primary measures of perceived functional health [28–30].

Our primary cognitive outcomes were domain latent factor scores constructed from a comprehensive cognitive test battery constructed to sample from five cognitive domains: Verbal Memory, Visuospatial Processing, Simple Attention, Set Maintenance and Shifting, and Reasoning. A trained psychometrician administered the battery listed in S1 Table.

### Statistical Methods

All analyses were performed on the ITT and PP cohorts. We first tested group differences in cardiorespiratory fitness and functional ability outcome measures. For repeated measures (LLFDI, SF-36, PPT) we used mixed-effects models (SAS-9.4, PROC MIXED; Group + Time + Group-by-Time). For change scores (%Δ in VO_2_ peak) we used ANOVA (PROC-GLM). In cases where we detected group differences in these omnibus tests, we used a nested contrast analysis to characterize patterns in the group differences [31]. We posited that group differences would fit one of three patterns: 1) *Practice Effect*, equivalent improvement across ALL groups; 2) *Intervention Effect*, equivalent improvement across exercise dose (Control <75min/wk = 150min/wk = 225min/wk); 3) *Linear Dose-Response*, linear improvement across exercise dose (Control<75min/wk<150min/wk<225min/wk). Because of the pilot nature of the study and the broad consensus that exercise is systemically beneficial [3] we used 1-tailed tests (α = 0.05).

For cognitive outcomes, we tested latent factors using a well-validated [32] multistep structural equation model of latent residual scores (SEM-LRS) [33]. These models provide purer estimates of a given cognitive ability than raw test scores and isolate variance associated with specific cognitive domains. Use of composite cognitive domain scores has been suggested as a powerful alternative to raw scores [34]. Analyses were conducted in PROC CALIS with direct estimation using full information maximum likelihood. SEM-LRS uses the variance and covariance of multiple tests to predict group means and standard errors. (See S1 File for further description.)

Lastly, we explored the relationship of changes in cardiorespiratory fitness and cognitive outcomes. We used longitudinal mediation analysis [35] to generate causal inferences about the relationship between cardiorespiratory fitness and cognition [36]. This approach specifically tested whether our experimental variable dose (duration of exercise in total minutes) drove cognitive change (latent residual score of a cognitive domain) directly or through cardiorespiratory adaptation (%Δ in VO2peak).

## Results

**Fig 1:**
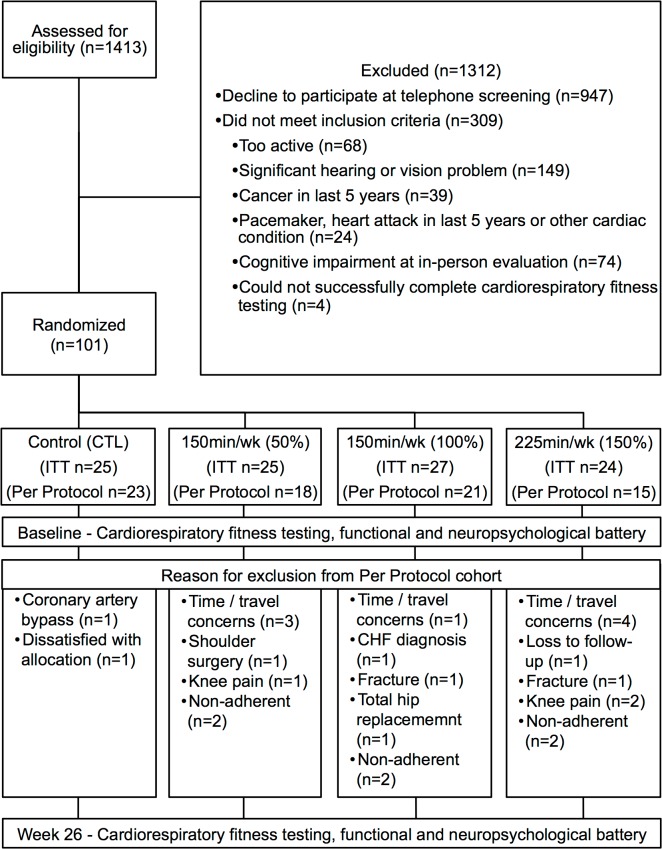
Study enrollment flow. Baseline measures of enrolled participants are provided in Table 1. Of the intent-to-treat (ITT) cohort (n = 101), 8 individuals withdrew due to time or travel concerns, 8 withdrew due to medical issues, 1 was dissatisfied with his group allocation, and 1 was lost to follow-up. Another 6 individuals were non-adherent to the exercise prescription. Those who did not adhere had slightly more education (17.8yrs [3.2] vs 16.1yrs [2.4]) otherwise there were no significant differences. The remaining 77 individuals were included in per-protocol (PP) analyses: control (n = 23), 75min/wk (n = 18), 150min/wk (n = 21), and 225min/wk (n = 15).

Of a potential 1,413 individuals who contacted the University of Kansas Alzheimer’s Disease Center while the trial was active a total of 101 participants elected to participate, met criteria, and were randomized to one of the four intervention arms: no change control (n = 25), 75min/wk (n = 25), 150min/wk (n = 27), and 225min/wk (n = 24) of AEx. This recruitment yield of 7% is similar to other exercise trials [18]. Most participants who contacted the center were uninterested or did not return follow-up contact (n = 947) after completing an initial phone screen. Others were medically ineligible (n = 241), primarily because of significant hearing or vision problems or too physically active to participate (n = 68). See Fig 1 for enrollment flow. Baseline measures of enrolled participants are provided in Table 1.

**Table 1:** Demographic and descriptive baseline data.

	Control (n = 25)	75min/wk (n = 25)	150min/wk (n = 27)	225min/wk (n = 24)
Age (yrs)	72.5 (5.8)	73.5 (5.9)	72.5 (5.7)	73.2 (5.3)
Education (yrs)	16.6 (2.4)	16.1 (2.8)	16.7 (3.4)	16.6 (2.2)
% Female (n)	64.0 (16)	63.0 (16)	63.0 (17)	66.7 (16)
**Cardiorespiratory Fitness and Physical Function**				
VO_2_ peak (ml/kg/min)	21.7 (4.2)	22.4 (4.1)	21.8 (4.3)	21.0 (4.5)
Total Minutes Exercised	NA	1595 (481)	3109 (808)	3562 (1812)
% of Prescribed Minutes Exercised / Week	NA	82.4 (24.7)	85.5 (21.3)	70.1 (32.5)
Physical Performance Test	32.0 (2.2)	31.8 (3.6)	32.3 (2.2)	31.9 (2.6)
Late-Life Disability Frequency Total	52.4 (4.6)	53.8 (5.8)	55.7 (4.9)	53.4 (3.6)
Late-Life Function Total	69.2 (9.0)	66.6 (9.7)	68.7 (7.5)	70.8 (8.2)
SF-36 Physical Component	56.4 (6.7)	54.2 (9.1)	56.4 (5.5)	57.7 (6.2)
SF-36 Mental Component	43.6 (4.2)	42.7 (5.1)	41.9 (3.9)	41.3 (5.0)

All values are group mean (standard deviation). LLFDI and SF-36 n = 100 due to computer malfunction. Baseline scores on component cognitive tests can be found in S2 Table.

### Intervention Adherence

We indexed adherence as minutes exercised as a percent of the total prescribed, accounting for the gradual build-up to their final dose (75min/wk = 1935min, 150min/wk = 3638min, 225min/wk = 5085min). In the ITT cohort, there were no differences in adherence across groups (p = 0.13) with the 75min/wk group completing 82.3% (1595 minutes), the 150min/wk completing 85.5% (3109 minutes), and the 225 min/wk group completing 70.1% (3562 minutes) of their prescribed exercise duration. The PP cohort excluded individuals who did not achieve over 80% of their prescribed exercise minutes. These individuals were generally fully adherent to the exercise prescription, achieving over 95% of their prescribed dose with no difference across groups (p = 0.9).

### Cardiorespiratory Fitness and Functional Health Outcomes

We observed a dose-response effect of AEx on cardiorespiratory fitness in the ITT cohort with linearly increasing gains in cardiorespiratory fitness (%Δ in VO_2_ peak) across dose groups: control = -4.4%, 75min/wk = 6.8%, 150min/wk = 7.7%, 225min/wk = 9.9% (Table 2). This dose-response was also observed in the PP cohort: control = -4.4%, 75min/wk = 6.4%, 150min/wk = 8.7%, 225min/wk = 11.0%. In ITT analyses, improvement in perceived disability was observed as a function of exercise dose (LLFDI Disability Total score: control = 0, 75min/wk = 0.7, 150min/wk = 1.3, 225min/wk 2.3), but this was not seen in the PP analysis. There were no observed changes in physical function (Physical Performance Test), perceived function (LLFDI Function Total score) or perceived physical and mental health (SF-36 composite scores) across dose groups.

**Table 2:** Mean fitness and physical function change from baseline in both the intent-to-treat and per protocol cohorts.

	26-Week Change									Hypothesis Testing	
	Control (n = 25)		75 min/wk (n = 25)		150 min/wk (n = 27)		225 min/wk (n = 24)			Group * Time Interaction	Best Fitting Model
**Intent-to-Treat Cohort (N = 101)**											
VO_2_ peak (% change)	-4.4	(7.1)	6.8	(7.6)	7.7	(7.1)	9.9	(9.1)		F(3,89) = 14.2, **p<0.001**	**Dose-response** t(89) = 3.8, p<0.001
VO_2_ peak (ml/kg/min)	-1.0	(1.6)	1.4	(1.5)	1.7	(14)	2.0	(1.9)		F(3,89) = 16.2, **p<0.001**	**Dose-response** t(89) = 4.0, p<0.001
Physical Performance Test	0.2	(1.7)	0.1	(2.0)	-0.9	(1.9)	0.5	(2.3)		F(3,133) = 1.4, p = 0.13	-
Late-Life Disability Frequency Total	0.0	(2.0)	0.7	(4.2)	1.3	(3.9)	2.3	(1.1)		F(3,123) = 3.1, **p<0.015**	**Dose-response** t(123) = 1.8, p = 0.015
Late-Life Function Total	-0.9	(9.2)	-0.3	(4.6)	0.0	(5.1)	-0.4	(7.9)		F(3,117) = 0.3, p = 0.43	-
SF-36 Physical Component	-0.3	(6.5)	-0.3	(4.6)	0.5	(6.1)	-0.8	(7.1)		F(3,125) = 0.3, p = 0.41	-
SF-36 Mental Component	-1.5	(4.5)	-0.2	(15.9)	-0.2	(4.0)	-0.4	(5.9)		F(3,153) = 0.3, p = 0.42	-
**Per-Protocol Cohort (N = 77)**											
	**Control (n = 23)**		**75 min/wk (n = 18)**		**150 min/wk (n = 21)**		**225 min/wk (n = 15)**			Group * Time Interaction	**Best Fitting Model**
VO_2_ peak (% change)	-4.4	(7.1)	6.4	(6.7)	8.7	(7.4)	11.0		(8.9)	F(3,76) = 15.8, **p<0.001**	**Dose-response** t(76) = 4.0, p<0.001
VO_2_ peak (ml/kg/min)	-1.0	(1.6)	1.4	(1.6)	1.9	(1.5)	2.4		(1.9)	F(3,76) = 17.9, **p<0.001**	**Dose-response** t(76) = 4.2, p<0.001
Physical Performance Test	0.1	(1.7)	0.1	(2.0)	-0.5	(2.0)	-0.1		(1.5)	F(3,114) = 1.0, p = 0.21	-
Late-Life Disability Frequency Total	0.0	(2.0)	1.4	(3.2)	1.0	(4.2)	1.9		(5.3)	F(3,96) = 2.0, p = 0.06	-
Late-Life Function Total	-0.9	(9.2)	0.0	(4.9)	0.7	(4.7)	1.0		(9.2)	F(3,98) = 0.9, p = 0.20	-
SF-36 Physical Component	-0.3	(6.5)	-0.1	(5.2)	2.0	(4.9)	1.1		(4.8)	F(3,92) = 1.3p = 0.15	-
SF-36 Mental Component	-1.5	(4.5)	-0.1	(6.2)	-0.8	(4.2)	-1.2		(5.1)	F(3,122) = 1.3, p = 0.14	-

The Group * Time interaction tests for group differences in response to exercise. The Best Fitting Model was assessed using an orthogonal contrast to test the shape of the dose-response. Both the interaction and the contrast had to reach a level of significance to be adopted as the best fitting model. In the ITT cohort, eleven individuals did not return for follow-up physical function testing and an additional six refused follow-up cardiopulmonary exercise test. Nine individuals did not return for follow-up cognitive testing. All values mean (standard deviation) unless otherwise noted.

### Cognitive Outcomes

We first tested the validity of our structural equation modeling of latent residual scores. Measurement models indicated the 5 derived cognitive domains were identically configured and loaded consistently onto the domain factors at baseline and 6-month follow-up (S3 Table and S4 Table detail ITT and PP latent factor construction, and latent factor and component subtest change scores.)

**Fig 2:**
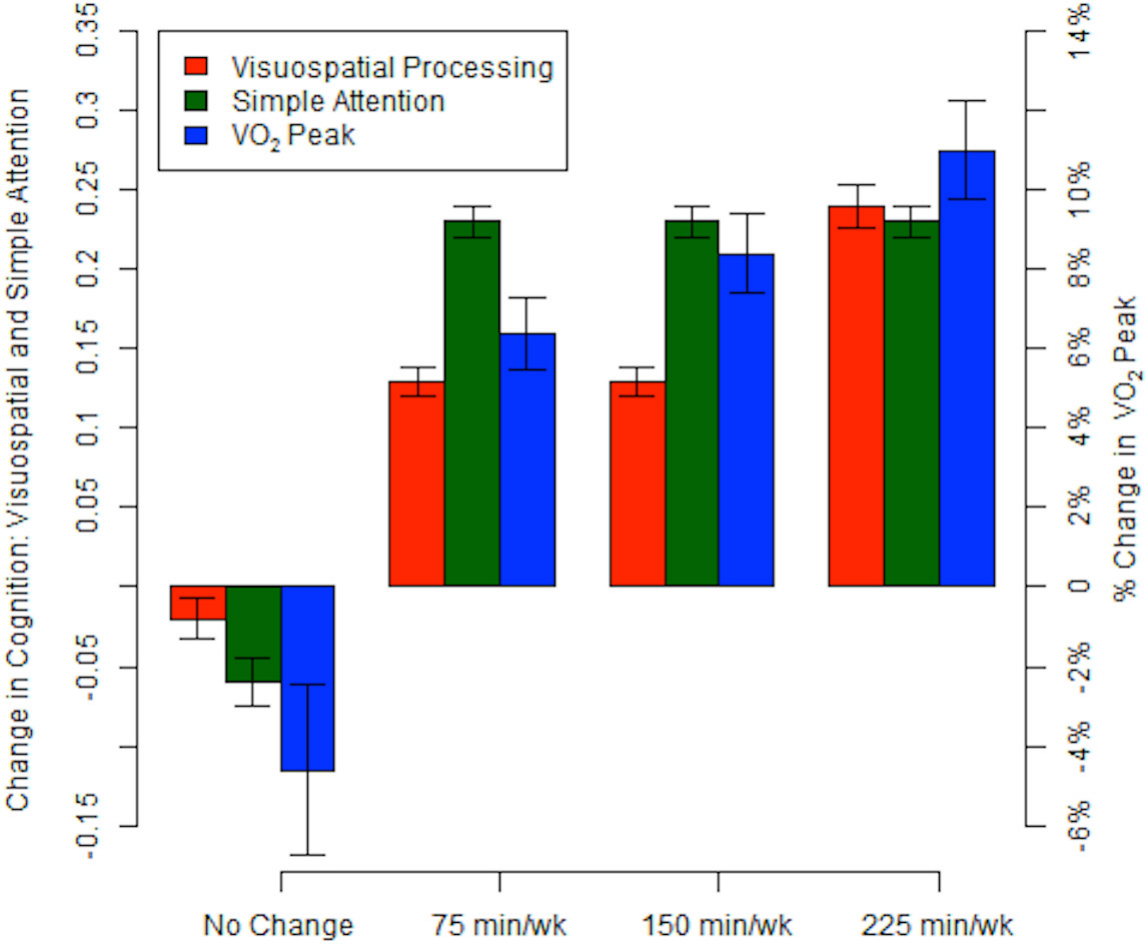
Visuospatial processing but not attention increases with increasing aerobic exercise dose. Percent change in VO_2_ peak (blue bars) increases in a dose-response fashion across the PP exercise groups. The best fitting model of Visuospatial Processing (red bars) follows a similar dose-response pattern. The best fitting model of Simple Attention (green bars) shows that any exercise results in improvement.

In ITT analyses, AEx was not associated with gains in any cognitive domain. In PP analyses Visuospatial Processing and Simple Attention improved with 6-months of any exercise. (Fig 2; S5 Table lists latent residual change score tests for all domains.) Simple Attention improved in all exercise groups equivalently indicating an intervention effect (ΔX^2^ = 22.0). Visuospatial Processing improved in a dose-response like function across the four doses of exercise, although the 75min/wk and 150min/wk groups were similar (ΔX^2^ = 18.0). The Verbal Memory (ΔX^2^ = 29.8) and Reasoning (ΔX^2^ = 34.1) domains also improved from baseline; however, all groups including controls improved equivalently, indicating a practice effect.

### Cognition and Cardiorespiratory Fitness Relationship

**Fig 3:**
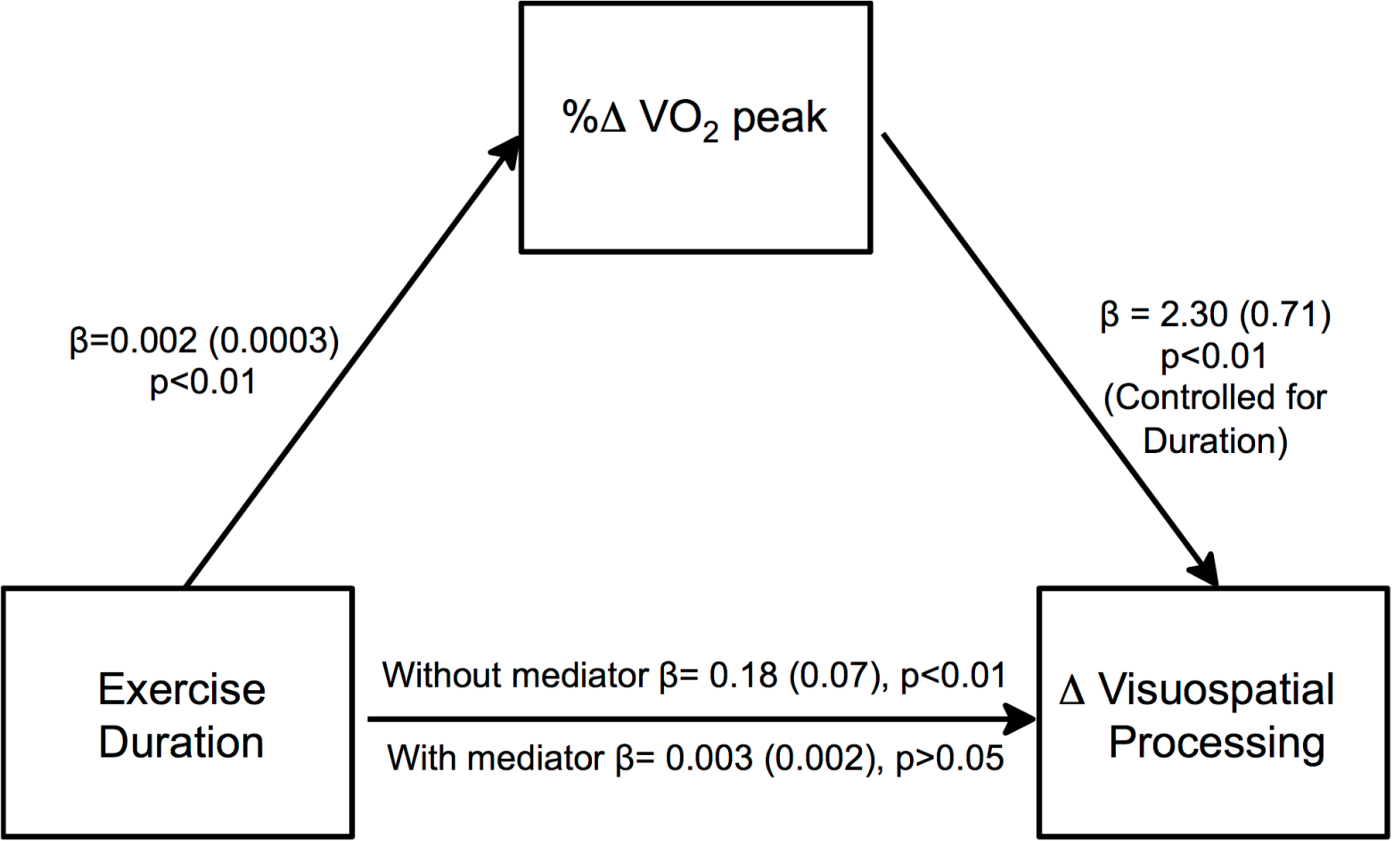
Cardiorespiratory fitness change mediates exercise duration effects on visuospatial performance. In the basic model without cardiorespiratory fitness change (%change in VO2 peak over 26 weeks) as a mediator, total number of minutes exercised (Exercise Duration) was associated with change in Visuospatial Processing. When change in cardiorespiratory fitness was added to the model as a potential mediator, it fully mediated the relationship of Exercise Duration and Visuospatial Processing improvement. Betas (Standard Error) are reported as the product of simultaneous regression with bootstrap replacement.

To clarify whether cognitive gain in the PP group was mediated by cardiorespiratory fitness, we performed mediation modeling. In the basic model, exercise duration (minutes exercised over 26 weeks) was correlated with change in Visuospatial Processing (Fig 3, p<0.05). If this relationship was due to another variable, such as cardiorespiratory fitness improvement, the significant relationship observed in the basic model should disappear when the candidate mediating variable was added to the model. Indeed, change in cardiorespiratory fitness (%Δ in VO_2_ peak) fully mediated the dose-response relationship between exercise duration and changes in Visuospatial Processing, rather than exercise duration alone.

### Adverse Events

There were 94 AE possibly or definitely-related to the intervention or VO_2_ peak testing: 86 (91%) mild, and 8 (9%) moderate severity. There were no severe AEs. Within the 3 exercising groups, AE were more common (X^2^[2] = 11.3, p = 0.003) in individuals exercising at 150min/wk (n = 40, 35 mild) and 225 min/wk (n = 36, 34 mild) than 75 min/wk (n = 15, 15 mild). Common mild AE related to the intervention included low back, hip, knee or foot pain. Moderate severity AE included lower extremity pain (n = 4), heart rhythm abnormalities (n = 3), and chest pain (n = 1).

## Discussion

There are three primary results from this pilot study. 1) Analyses restricted to individuals adhering to and completing the study suggest that visuospatial and attention benefits may be attained at low doses of exercise with visuospatial benefits appearing to increase with increasing exercise dose. 2) There is a clear dose-response effect of AEx on cardiorespiratory fitness for older adults validating our community-based protocol for the delivery of a rigorously controlled exercise dose. 3) In mediation analyses, the apparent cognitive benefits of AEx are best explained by gains in cardiorespiratory fitness, suggesting that prescribing individualized exercise to maximize cardiorespiratory fitness may be important for realizing exercise-related cognitive benefits.

Our finding of a dose-response relationship between exercise and cardiorespiratory fitness replicates the findings from a randomized dose-response study in post-menopausal women [18]. Cardiorespiratory fitness is an important health measure, strongly linked to mortality, cardiovascular disease risk, functional health, and cognitive decline [3]. In older adults, cardiorespiratory fitness declines >2% a year [37]. Our data demonstrate that even modest levels of exercise in older adults provide fitness benefits, with increasing physiologic benefits at higher doses. These data reinforce the idea that systemic physiologic adaptations to exercise are well-preserved in older adults, even at low doses of AEx, and support the general consensus that greater benefits are achieved at higher doses of exercise. These findings also validate the rigorous delivery of varying doses of exercise through a community-based semi-supervised exercise program. Further, our results suggest achieving a modest goal of 75 min/wk of AEx provides benefits to cognition and perceived health, consistent with epidemiological data that high exercise levels are not necessary to achieve cognitive benefits [7].

The finding that cardiorespiratory adaptation predicts cognitive benefit may indicate that cardiorespiratory fitness (or aerobic capacity) is a useful therapeutic target for achieving cognitive benefits. If so, exercise prescription should be individualized to impact cardiorespiratory fitness rather than simply prescribing a standard amount of physical activity. Health care providers and exercise professionals can assist individuals following standard principles of exercise prescription, including varying frequency, intensity, time, and type of activity to maximize cardiorespiratory gain. Our finding that duration of exercise may be less important than the physiological cardiorespiratory fitness response also underscores prior work suggesting that subpopulations of non-responders with genetic or physiologic limiting factors may exist [38], warranting further investigation into predictors of exercise response.

We applied longitudinal modeling techniques to measure domain-specific cognitive changes. In the ITT cohort, we found no effect of exercise on cognitive performance. However, when we narrowed our analysis to those who exercised per-protocol, we found Simple Attention improved over 26 weeks for all exercise groups (but not in a dose-dependent manner) compared to controls and Visuospatial Processing improved non-linearly with increasing exercise dose. Our Visuospatial Processing factor is largely dependent on executive function, the cognitive domain that prior exercise studies have suggested most responds to exercise. Executive function is a loosely-defined concept that is dependent on selective attention, speed of processing, and visuospatial information. It is therefore likely that both our Attention and Visuospatial Processing domains reflect core features of executive function. We previously identified this Visuospatial Processing domain as one that declines in the years prior to the onset of dementia [39]. Our finding that participants perceived dose-dependent improvement in disability (LLFDI) suggests gains in visuospatial function and attention may also be associated with real-world benefits.

This study has a number of notable strengths and limitations. Because this study was designed as a pilot trial to inform the development of more definitive trials, the sample size and intervention duration of 6-months are modest. Our control group had no sham activity and thus we are unable to control for psychosocial effects. We used intensive clinical methods (Clinical Dementia Rating) and a comprehensive cognitive testing battery to carefully exclude individuals with subtle cognitive impairment. As exercise intensity was standardized across groups, we have no direct data on the importance of exercise intensity, an important determinant of cardiorespiratory adaptation [3], in mediating cognitive benefits. Finally, exercise was semi-supervised after the first 6 weeks and thus at times exercise time and intensity was self-reported. Nevertheless, our community-based approach enhances the generalizability and ecological validity of the findings and we rigorously controlled the intervention across personal trainers and facilities through intensive training and monitoring to minimize variability in protocol delivery. Despite this, it was difficult for the 225/wk group to achieve their exercise goals. It may be that the rigid structure of a randomized controlled trial (e.g. controlled duration, exercise modality) impedes adherence at higher doses. Anecdotally, there were more reports of boredom in this group. While our results argue that this dose level is feasible for many, it remains to be seen if it is the most efficacious dose.

This 26-week pilot randomized controlled trial demonstrates a clear dose-response effect of AEx on cardiorespiratory fitness and suggests that a dose-response may be present for exercise-related cognitive benefits. Our data suggest that high levels of exercise are not necessary to achieve fitness and cognitive benefits in those who adhere to the exercise program, supporting the simple clinical directive that ‘any exercise is good, more is better’. Importantly, we found that the presence and degree of physiologic adaptation to AEx (i.e., increased VO_2_ peak) is an important predictor of cognitive benefit. In fact, a physiologic response to exercise was a better predictor than exercise dose (total duration of exercise) in predicting cognitive benefits. This suggests that maximizing an individual’s cardiorespiratory fitness may be an important therapeutic target to achieving visuospatial cognition and attention benefits. Public health efforts aimed at initiating and maintaining any dose of exercise remain important [40] although greater benefits are likely to be achieved with higher doses and through achieving gains in cardiorespiratory fitness.

## Supporting Information

S1 File Supporting methods. (DOCX)

S2 File Research protocol. (PDF)

S3 File CONSORT checklist. (PDF)

S1 Table Description and baseline performance on component cognitive tests. (DOCX)

S2 Table Weekly progression of exercise duration in minutes. (DOCX)

S3 Table Table of log-likelihood values for SEM-LRS models showing the 5 derived cognitive domains. The table shows that the 5 derived cognitive domains were identically configured and loaded consistently onto the domain factors at baseline and 6-month follow-up.
(DOCX)

S4 Table Standardized change scores for the latent and component subtest scores in the Intent-to-Treat (n = 101) and Per-Protocol Cohorts (n = 77). Values are normalized mean and standard error (M = 0, SE = 1). Each value represents change in the group over 26 weeks.
(DOCX)

S5 Table Group latent factor score estimates, best fitting trend models, and change in cognitive domain subtest Z-scores (M = 0, SE = 1) in the Per-Protocol Cohort. Data represent mean change (SE) in cognitive domain latent factors over 26 weeks (unconstrained latent mean change scores [M = 0, SE = 1]). *Hypothesis Testing*: If differences were present at the group level in 26-week cognitive outcomes, we used a nested contrast analysis to characterize whether those differences fit one of three patterns: (1) Practice Effect, equivalent improvement across all groups; (2) Intervention Effect, equivalent improvement across exercise doses; (3) Linear Dose-Response, linear improvement across exercise doses. The most parsimonious models accepted by the nested comparison procedures are reported in bold. Degrees of Freedom (df) for SEM models are the difference between the number of parameters estimated in unrestricted structural model and the number of parameters estimated in hypothesis driven (constrained) model. ∅ indicates that the trend model did not fit. * 26-week change scores for Set Maintenance & Shifting were different than zero. However, nested contrast testing failed to reach significance indicating no distinct pattern of change from baseline.
(DOCX)
