# Supplementary material for: Dose-Response of Aerobic Exercise on Cognition: A Community-Based, Pilot Randomized Controlled Trial
Source: PLoS One. 2015 Jul 9;10(7):e0131647. doi: 10.1371/journal.pone.0131647 (PMC4497726; doi:10.1371/journal.pone.0131647)
Supplement: S1 File — (DOCX) [file pone.0131647.s001.docx]

**S1. Supporting Methods**

***Structural Equation Modeling Detailed Description***

The structural equation model of latent residual scores (SEM-LRS) is a logical progression of hierarchical hypothesis testing of means and variances conducted in a step-like fashion to investigate how well a given model fits the observed data (S3 Table). The goal is to create a parsimonious composite of contributing tests that uniquely capture a hypothesized domain, defined by common variance shared across subtests (S4 Table). The level at which model constraints fail to fit informs the hypothesis testing during model building process. If the comparison of the less and more restricted models is “invariant” (i.e., the additional constraints fit the variance-covariance structure observed in the data) then the constraints added by the more restricted model are adopted and the next step in the SEM-LRS can be conducted (more constraints added). If not, then the additional constraints fail to fit the data and the constraints are rejected and alternative constraints must be tested. Final models are adopted when the investigator exhausts all options available for constraining a SEM-LRS at any given level (prescribed by a hierarchy of possible/allowable logical constraints). Thus the iteratively developed final model is the most constrained (parsimonious) model that still explains significant variance in the observed data (empirically valid).

The SEM-LRS requires the development of first a measurement model and then a structural model. The measurement model is a mapping of observed test performance onto a latent cognitive domain (e.g., What are potent indicators of the Verbal Memory domain?). The resultant cognitive domain is defined by the variance shared among its multiple indicators. The confirmed measurement models from the previous step are then applied in a repeated measures SEM to predict the latent scores at 6-month follow up, residualized after controlling for baseline performance. This structural model informs us how a cognitive domain changes over time (e.g., What is the effect of baseline Verbal Memory performance on follow up performance?) or how the groups differ (e.g., Is Verbal Memory performance for the any exercise group different then the control group?). The structural model tests relationships among latent variables (Baseline vs. Follow up or Group 1 vs Group2) and is conducted by a set of prescribed hierarchical hypothesis tests for the structural model. Because the measurement modeling process is intended to create a valid longitudinal model of domain-specific performance we used the ITT (n=101) sample in this phase of model building. Because the structural modeling process is intended to test study predictions we used the Per-Protocol cohort (n=77), but also report ITT omnibus tests for reference (S5 Table).

**Steps to validate the measurement model (N = 101; S3 Table):**

Step 1. Repeated measures (RM) configural modeling of the intent-to-treat data for each of the 5 cognitive domains verified that expected subtests contributed significantly to hypothesized domains (Verbal Memory, Visuospatial Processing, Attention, Set Maintenance and Shifting, and Reasoning). This step verified that selected component subtests share sufficient variance to warrant labeling the common variance component at both baseline and follow up times of assessment with the same label (e.g., Verbal Memory).

Step 2. RM weak invariance model verifies that the subtests’ regression coefficients contributing to the latent variable at baseline were similar to the regression coefficients at follow up.

Step 3. RM strong invariance model verifies that the indicator intercepts at baseline and follow up were similar (i.e., observed measures should have statistically equivalent intercepts at each time-of-assessment when both latent variables are 0).

**Steps to validate and test the structural model (n = 77; S4 Table):**

To test the null hypothesis that cognitive scores would not change from baseline, we used an identical strategy to test the multivariate structural models as we did for the univariate regression models; however, we applied the SEM-LRS strategy and used a series of -2LL to test goodness-of-fit for nested models in place of univariate *F*-tests.

**Step 1- Validating a baseline structural model.** A good-fitting SEM-LRS constrained RM regression model (CFI > .90) verifies that the effect of baseline performance on follow up (autocorrelation) is equivalent across groups; however, all latent means and variances are free to vary. Because this model is direct re-specification of the RM strong invariance measurement model (S1 Table), all -2LL values are identical; however, this model of autocorrelation can be used as a baseline to compare the goodness-of-fit for the null hypothesis test and follow up contrasted regressions.

**Step 2 - Hypothesis test of latent means.** Rejection of the null required two Δ-2LL goodness-of-fit tests: A protected test of the Group X Time interaction and a dose-response contrast. We tested if there was any change in latent residual scores across time (equivalent to the Group X Time interaction in the univariate testing strategy) by comparing a structural model with all constraints free to vary (baseline described in Step 1) against a model where the latent means were all constrained to zero (0 =M1 = M2 = M3 = M4; in all tests of the structural model the latent variances were all constrained to be equal). To reject the null hypothesis, the additional equivalence constraints had to result in significantly poorer fit than baseline. Only if we rejected this all means equal zero model, did we then test whether the profile of latent residual scores conform to any of the 3 *a priori* hypothesized shapes.

**Step 3 - Follow up contrasts (S5 Table).** In the case that latent means were not all equal to zero we then described the shape of Group X Time differences across dosage using a similar set of planned contrasts specified in the univariate tests: Practice effect, exercise effect, or a dose-response effect (in all cases we allowed latent variances to vary freely).

**Step 3a. - Practice effect** (0 < M1 = M2 = M3 = M4). All groups equal at follow up (e.g., Are all of the groups’ Standard Deviations of Verbal Memory factor scores the same?).

**Step 3b. - Intervention effect** (0 = M1 < M2 = M3 = M4). Asymptotic effect for exercise dose at follow up (e.g., Do all of the groups who exercised perform better on Verbal Memory irrespective of dose?). All exercise doses result in equivalent increases and exercise is greater than no exercise controls.

**Step 3c. – Dose Response** (0 = M1 ≤ M2 ≤ M3 ≤ M4). A dose-dependent treatment response across arms (equivalent to the dose-response contrast in the univariate testing strategy) where latent means were constrained to conform to a monotonic increasing function (M1 = 0 ≤ M2 ≤ M3 ≤ M4).
