# Supplementary material for: Dose-Response of Aerobic Exercise on Cognition: A Community-Based, Pilot Randomized Controlled Trial
Source: PLoS One. 2015 Jul 9;10(7):e0131647. doi: 10.1371/journal.pone.0131647 (PMC4497726; doi:10.1371/journal.pone.0131647)
Supplement: S2 File — (PDF) [file pone.0131647.s002.pdf]

RESEARCH DESIGN AND METHODS

Study Design

This is a randomized, 26-week study of supervised exercise assessing three doses of exercise in sedentary individuals 75 years of age and over with age-related cognitive changes. A total of 100 subjects (n=25 in each of four groups) will be randomized to non-aerobic control, 50%, 100%, or 150% of the recommended exercise dose for 26 weeks of supervised exercise (3 or 4 days a week), per the DREW protocol.[115] Participants will be directly supervised during all exercise sessions for the first two months after which direct supervision will occur during at least one session a week. This is intended to provide increased flexibility while also maintaining direct contact with the participant to enhance adherence. Aerobic fitness, physical function, and cognition will be assessed at baseline and 26 weeks to examine the dose-response relationships. At 52 weeks, cognition will be reassessed to examine durability of cognitive changes.

Timeline: We anticipate beginning enrollment within the third month of the grant. We project meeting our enrollment goal within 21 months of study initiation (18 months of active enrollment) with the last enrolled cohort of subjects completing their 26-week intervention in the first quarter of the third year. This will allow time to complete 52-week cognitive assessments on the latest subjects enrolled. An enrollment goal of approximately 5 subjects a month is feasible and realistic given our history of recruiting individuals for investigator-initiated and ADCS-sponsored trials. Data analyses will occur in the second half of the third year along with preparation of reports, presentations and manuscripts.

TIMELINE	Year 1	Year 2	Year 3
Recruitment / Enrollment			
Intervention			
Data Analyses			
Manuscript preparation			

Rationale for Aerobic Exercise Dose and Duration

We have chosen to examine aerobic exercise, as opposed to resistance exercise, given the accumulating data suggesting aerobic fitness is related to brain health.[2, 64] Aerobic exercise is a mainstay of all public health recommendations. We suspect that walking at moderate intensity is the most common form of exercise for older adults and is easily adopted and widely prescribed. Thus, it likely has the most relevance to public health recommendations. The current study design attempts to isolate aerobic exercise and related variables (such as VO2 peak) in order to examine their role in enhancing cognition while examining fundamental questions regarding the dose-response of exercise on important exercise-related cognitive and physiologic outcomes.

Exercise Dose: The three doses of aerobic exercise were chosen as they approximate 50%, 100% and 150% of the current public health recommendations and thus have important practical, “real-world” implications. The aerobic exercise study intervention and dosing calculations are based on proven methods that have been tested in a cohort of post-menopausal women age 45 - 75.[88, 115] Target energy expenditure (expressed in kcal/kg/week, KKW) is based on the current public recommendations for 30 minutes of exercise at moderate intensity 5 days a week. using methods based on the DREW study.[115] Thus, we will assess whether cognitive and physiologic benefits are present at minimal exercise doses (i.e., 50%) and if increasing doses are associated with additional gains.

Based on our preliminary data in older adults 75 and over (n = 41) with a mean VO_2^{peak} of 19.3 ml/ kg/ min and target intensity of 50% of VO_2^{peak}, participants would expend 7KKW to meet the current public health recommendations of 30 minutes of exercise five days a week. [50% of mean VO_2^{peak} = 9.65 ml/ kg /min converted, per the DREW protocol,[115] to metabolic equivalents (METs, 3.5 kcal/kg/hr) is equal to 1.39 kcal over 30 minutes; 1.39kcal * 5 days = 7KKW]. Energy expenditure group doses set at 50%, 100% and 150% are therefore 3.5, 7 and 10.5KKW. As in DREW, all groups will exercise at the same intensity: 50% of the VO_2^{peak} calculated as 50% heart rate reserve based on the Karvonen formula.[116]

In the DREW study, individuals in the 50% dose group exercised an average of 72.2 minutes per week. In the 100% dose group, weekly exercise duration was 135.8 minutes and 191.7 minutes in the highest dose group. Adherence was high (>89% in all groups) and not influenced by age and comorbidities. Thus, in adhering to the DREW protocol, exercise dose targets are achievable through moderate (50% VO_2^{peak}) exercise such as brisk walking 3-4 days per week.[115,117]

Exercise Duration: The intervention length of 26 weeks was chosen as substantial physiological adaptations to exercise occur in the first 3 to 4 months of exercise and major changes are unlikely beyond 6 months of training.[115] Considering the increased logistical and participant burden of longer durations, we chose a 26-week intervention for this pilot study. We include a 52-week cognitive assessment to examine

durability of any observed cognitive changes. Additionally, we will assess physical activity levels at 52-weeks to assess sustained behavioral changes of increased physical activity after the end of the intervention.

Patient Population

Sample: The greater Kansas City bi-state area (Missouri and Kansas) is home to 2.3 million people with 2004 estimates indicating over 11% of the population is 65 years and older. Nearby cities include Lawrence, Topeka, and Atchison in Kansas and St. Joseph, Warrensburg, and Chillicothe in Missouri. Enrolled subjects will be sedentary adults with age-related cognitive decline aged 75 and older. The rationale for focusing on adults over the age of 75 is related to the increased cognitive and functional decline in this rapidly growing population.[19] To our knowledge, an exercise dose-response study has not been done on this population. Further defining the role of exercise in attenuating age-related decline in physical and cognitive function in this age-group would have important public health implications.

**Inclusion Criteria**	**Exclusion criteria**:
• Informed consent<br>• Age 75 years or older<br>• Sedentary except for casual recreation such as bowling, shuffleboard, billiards etc. Sedentary will be defined as < 500 kcal per wk of exercise physical activity as estimated by the Minnesota Leisure Time Physical Activity Questionnaire.[97]<br>• Memory Complaints<br>• Telephone Interview of Cognitive Status performance in "unimpaired" range<br>• Community dwelling with a caregiver willing to accompany the participant to visits to the screening evaluation. The caregiver must visit with the subject more than five times a week.<br>• Nondemented: CDR 0<br>• Adequate visual and auditory abilities to perform all aspects of the cognitive and functional assessments<br>• Stable doses of medications for at least 30 days prior to screening.<br>• Likely to participate in all scheduled evaluations and complete the exercise program over 52 weeks	• Dementia<br>• CDR > 0<br>• Current clinically significant major psychiatric disorder (e.g., Major Depressive Disorder) according to DSM-IV criteria or significant psychiatric symptoms (e.g., hallucinations) that could impair the completion of the study<br>• Current clinically-significant systemic illness likely to result in deterioration of the patient's condition or affect the patient's safety during the study<br>• History of clinically-evident stroke<br>• Clinically-significant infection within the last 30 days<br>• Myocardial infarction or symptoms of coronary artery disease (e.g., angina) in the last two-years.<br>• Uncontrolled hypertension within the last 6 months<br>• History of cancer within the last 5 years (except non-metastatic basal or squamous cell carcinoma)<br>• Hemoglobin less than 11 g/dL<br>• History of drug or alcohol abuse as defined by DSM-IV criteria within the last 2 years<br>• Insulin-dependent diabetes mellitus<br>• Significant pain or musculoskeletal disorder that would prohibit participation in an exercise program

Recruitment: We will draw on our experience in recruiting study participants from the Kansas City area. The KU Alzheimer and Memory Program has established a history of excellent recruitment and performance in investigator-initiated studies and ADCS-sponsored clinical trials. Pat Laubinger, our current recruitment coordinator, will have the primary responsibility of recruiting participants. Additionally, we have had success with cost-effective advertising locally on radio stations and on the internet (Google Adwords) with links to our website. We have several databases of potentially-recruitable subjects including a large phone inquiry databases. Additionally, the community-based YMCA network is likely to enhance recruitment and retention given the convenience of the locations across the Kansas City area. We are confident that we will meet our enrollment goals given our track record of excellent recruitment for studies (see Preliminary Results).

Randomization: Participants will be randomized equally to one of three groups: 3.5 KKW, 7 KKW, and 10.5 KKW. The randomization procedure will be stratified by gender to ensure the groups are well-matched on gender. Block randomization will be used defined by gender and the randomization sequences will be generated by computer software.

Study Blinding: Outcome assessments will be performed by blinded raters. Heather Anderson, MD, an experienced geriatric neurologist will be the primary rater for performing CDRs. Phyllis Switzer (psychometrician) will also be blinded to the participant's intervention group and will perform the psychometric assessments and administer the scales and questionnaires. Dr. Burns (PI) will be unblinded and perform safety assessments, review laboratory data, and address safety concerns or adverse events.

Study Procedures / Outcome Measures

Telephone Screen: After initial contact with the potential participant to provide information on the study, interested participants will first undergo a telephone screen designed to efficiently identify those who will clearly not meet inclusion and exclusion criteria. The telephone screen will consist of an assessment of self-reported memory complaints, self-reported medical history, physical activity level (to determine "sedentary" status) and the Telephone Interview of Cognitive Status (TICS).[118]

Memory complaints will be assessed using an efficient screen composed of two questions self-rated on a Likert scale.[119] Question 1 is "How often do you have trouble remembering things?" with responses ranging from 1 (never) to 5 (very often). Question 2 is "How is your memory compared to ten years ago?" with responses ranging from 1(much better) to 5 (much worse). Individuals whose combined score is 7 or higher (range 2 – 10) will be eligible for enrollment. A score of 7 was the median score in a sample of 90 individuals with a mean age of 87 years who came to autopsy. Higher subjective memory complaints were correlated with AD pathology, suggesting that this scale has biological relevance.[119]

The Minnesota Leisure Time Physical Activity Questionnaire[120] will be used to assess physical activity levels. Sedentary will be defined as < 500 kcal per wk of exercise physical activity estimated by the questionnaire. This level of activity includes only casual recreation such as bowling, shuffleboard, billiards etc.

The TICS is a brief, standardized test of cognitive functioning designed to be administered via the telephone and has excellent sensitivity and specificity for the detection of cognitive impairment. The test contains 11 test items and takes less than 10 minutes to administer. Participants who fall in the "unimpaired" range based on the normative sample of 6,388 subjects will be considered for an in-person screening evaluation.[118]

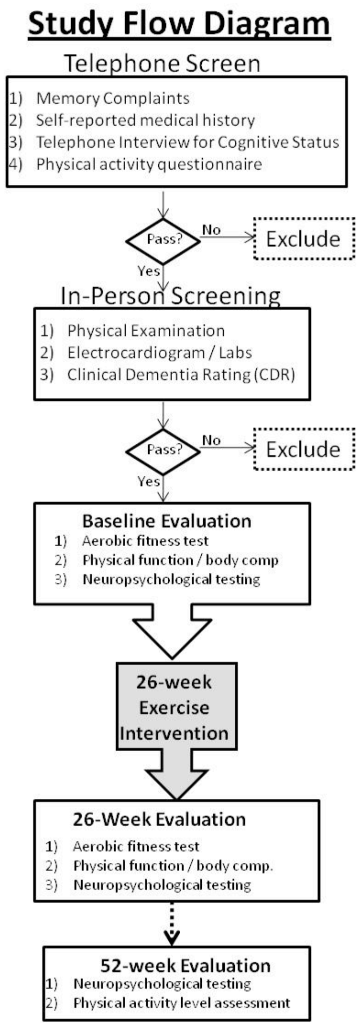

Medical history and medications will be reviewed with particular attention to a history of stroke, coronary artery disease (active in the last two years), insulin-dependent diabetes, orthopedic or mobility issues, psychiatric disorders, cancer, and recent history of drug or alcohol abuse.

In-Person Screening Evaluation: The primary purpose of the screening evaluation is to 1) obtain informed consent from the participant and, 2) to establish the participant meets inclusion and exclusion criteria. Medications, past medical history, education (years of formal education), demographic information, and family history will be collected from the informant by the nurse clinician. Clinical laboratory evaluations will be assessed for electrolytes (sodium, potassium, chloride, bicarbonate), renal function (creatinine, blood urea nitrogen), complete blood count, and hemoglobin.

A standard physical and neurological examination will be performed by a clinician. Vital signs will include heart rate, respiratory rate, weight, body temperature and blood pressure (two measurements of systolic and diastolic blood pressure made using a manual cuff on the left arm with the subject in a seated position). A 12-lead electrocardiogram (ECG) will be reviewed by a clinician.

The presence or absence of dementia is determined using the Clinical Dementia Rating (CDR).[121, 122] Only nondemented (CDR 0) subjects will be enrolled. The CDR is conducted by a clinician through semi-structured interviews with the participant and a collateral source knowledgeable about the participant. These methods have a diagnostic accuracy for AD of 93%.[123] Based on the collateral source and participant interviews, a global CDR score is derived such that CDR 0 indicates no dementia and CDR 0.5, 1, 2, and 3 represent very mild, mild, moderate, and severe dementia, respectively.

Baseline Evaluation: The purpose of the baseline evaluation will be to perform outcome assessments prior to initiating the intervention. The baseline evaluation will consist of 2 visits occurring within 30 days of initiating the exercise protocol. First, participants will be evaluated in the GCRC (3 – 4 hour visit) with a maximal exercise test to assess VO_2^{peak}, a DEXA scan for body composition, and physical function testing. Next, psychometric testing (2-hours) and other scales and questionnaires will be performed during visit 2.

Telephone Checks: Participants will be contacted by phone for formal review of medication changes, medical history, and adverse events within 3 days of initiating the exercise intervention and again at weeks 6,

12, and 18 during the active intervention. These formal assessments will complement the routine daily assessments performed by YMCA exercise instructors that briefly assess new medical or exercise-related adverse events. Additionally, the telephone checks will encourage communication between the study team and the participants and are partially designed as a way to encourage compliance with the intervention.

Given we will also be assessing durability of cognitive changes at 52-weeks, we will continue telephone checks at weeks 32, 39, and 46 weeks during the 26-week follow-up period after completion of the active intervention. These follow-up telephone checks will serve to enhance retention and encourage continued physical activity (in the active group).

26-Week Post-Intervention Evaluation: During the 26th week (+/- 7 days), participants will repeat the outcome assessments during two visits. Again, all participants will be evaluated in the GCRC with a maximal exercise test to assess VO2 peak , a DEXA scan for body composition, and physical function tests. Next, blinded raters will perform psychometric testing and scales and questionnaires.

52-Week Follow-up Evaluation: At 52 weeks, all participants will have the full battery of neuropsychological testing, scales, and questionnaires to assess durability of exercise-related changes. Additionally, the Community Healthy Activities Assessment Model in Seniors (CHAMPS) will be repeated to assess durability of changes in physical activity level outside of the supervised exercise program.

TABLE 2: STUDY CALENDAR

	Procedures	TSc	**V1**	**V2**	TC1	TC2	TC3	TC4	**V3**	TC5	TC6	TC7	**V4**
			Screen	Baseline									
			D -30	D-29to-3	D-3	W6	W12	W18	W26	W32	W39	W46	W52
	Informed Consent		X										
	Inclusion / Exclusion		X		X								
	Clinical Labs†		X										
Screening Procedures	TICS	X											
	Memory Complaints	X											
	Physical Activity	X											
	Medical History	X	X		X								
	CDR		X										
	Physical/ Neuro		X						X				
	12-lead ECG		X						X				
Safety Assessments	Vital Signs		X						X				
	Concurrent meds		X		X	X	X	X	X	X	X	X	X
	Adverse Events					X	X	X	X	X	X	X	X
	Aerobic Fitness (VO_2^{peak})			X					X				
	Neuropsych. Testing‡			X					X				X
Outcome Assessments	Scales / Questionnaires§			X					X				
	DEXA			X					X				
	Blood draw / banking			X					X				
	Physical Function£			X					X				
	CHAMPS			X					X				X

V: visit, D: day, W: week, TSc: Telephone screen, TC: telephone contact, CDR: Clinical Dementia Rating, DEXA: Dual energy x-ray absorptiometry for body composition, CHAMPS: Community Healthy Activities Model Program for Seniors for physical activity.

†**Clinical Labs:** Electrolytes (sodium, potassium, chloride, bicarbonate), renal function (creatinine, blood urea nitrogen), complete blood count, hemoglobin

‡**Neuropsychological Testing:** Hopkins Verbal Learning, Selective Reminding Test, Logical Memory, Boston Naming, Spatial Span, Visual Reproduction, Faces, Fluency, Stroop, Wisconsin Card Sorting, Trail Making A and B, Digit Span, Digit Symbol, Inductive and Matrix Reasoning, Picture Arrangement

§**Scales / Questionnaires:** Geriatric Depression Scale, SF-36, ADCS-Activities of Daily Living, Barthel Index,

£ **Physical function:** Physical Performance Test, Timed-Up-and-Go, and strength testing (grip and lower extremity).

Aerobic Exercise Procedures

The aerobic exercise intervention will begin within thirty days of completion of the screening and baseline evaluations. To improve safety and adherence to the protocol, participants will exercise under the close supervision of a certified trainer 3 – 4 days per week for 26 weeks in a local YMCA facility most convenient for the participant. Aerobic exercise will consist primarily of walking on a treadmill although participants can choose to exercise on a cycle ergometer one day a week to avoid boredom. As in the DREW study, participants will spread their exercise over 3 – 4 days per week. Exercise dose will be manipulated by exercise duration. Exercise intensity will be monitored and maintained at 50% of the VO_2^{peak} determined by treadmill testing. An initial run-up period will occur so that participants can safely achieve exercise levels at their group

prescription. All participants will begin exercising at the 3.5KKW level. For those in the 7 and 10.5KKW groups, target energy expenditure will be increased by 1KKW each week until the assigned level is achieved.

Exercise trainers will assist participants in adjusting exercise routines to achieve their energy expenditure goals. Use of equipment, achievement of target HR and safety will be closely monitored by the trainer through the course of the study. All sessions include 5-minute warm-up and cool-down periods. Each subject will wear a Polar F4 heart monitor (Polar USA) for recording heart rate during each exercise session. Subjects unable to exercise continuously on the treadmill will perform intermittent training until the target duration is reached. Aerobic fitness training logs will be maintained for heart rate and perceived exertion rating scales. Intensity levels will be monitored and recorded by exercise personnel on a daily basis by heart rate assessment and ratings of perceived exertion. Adherence will be followed and recorded by assessing the participant's attendance and ability to meet weekly energy expenditure goals.

Exercise Supervision: Direct supervision by exercise instructors will occur for all exercise sessions for the first 8 weeks, after which more flexibility may be introduced into the schedule if the participant is consistently and safely meeting their target expenditure. Participants will be allowed to perform unsupervised exercise sessions at the YMCA at times (i.e. early morning, nights, and weekends) when exercise trainers may not be available. The participants will still be required to have at least one directly supervised exercise session per week to maintain contact with program staff and encourage adherence to the program. Unsupervised exercise sessions will be conducted similarly to supervised exercise sessions with heart rate monitoring and logging of RPE and session details by the participant. Session data will be reviewed by the exercise instructor at the weekly supervised session. Participants will be encouraged, however, to perform as many exercise sessions as possible, in not all, under direct supervision.

Adherence and Retention*:* Exercise protocol adherence will be more directly monitored as the percentage of estimated weekly energy expenditure based on exercise time. If participation falls below adherence goals, participants will not be excluded from the study (given that our main analysis will be intent-to-treat) and we will work to further encourage participants and identify problems that may be barriers to exercise compliance. Should a patient (or caregiver) request or decide to withdraw, we will ask the participants to attend the 26-week and 52-week outcome assessments. All participants will undergo a formal, structured interview at the end of the intervention to provide feedback on the program and identify barriers and limitations to exercise adherence.

To enhance and encourage adherence, we have introduced some flexibility into the schedule. As noted above, after 8 weeks subjects will have the option to perform exercise sessions at times when instructors may not be available. On the other hand, we have balanced this flexibility with our experience that adherence is enhanced with direct contact with participants. Thus, we will continue to require at least one directly supervised exercise session a week to maintain participant contact in addition to allow close monitoring of the participants progress to the weekly exercise goal.

We also recognize and anticipate that participants may need to miss several exercise sessions due to travel plans, family obligations, etc. If missed sessions are anticipated, participants can increase weekly energy expenditure up to 1.5KKW each week to compensate for reductions in their dose. Additionally, a Polar RS400 Running Computer (Polar USA) can be issued to the participant to record exercise data if traveling and the only option is to exercise on their own. The Polar RS400 is worn as a watch during exercise and allows storage of data for multiple exercise sessions (as opposed to one session with the Polar F4). The subject will be asked to exercise by walking while maintaining their target heart rate to achieve their exercise duration goal. Participants will be strongly discouraged from making these accommodations more than 2 times over the course of the study.

A number of additional activities and incentives are planned to encourage retention. We have chosen to utilize the YMCA as it is community-based and thus reduces the travel needed to attend the exercise sessions, improving compliance and retention. The participant will receive a one-year family membership to the YMCA to encourage other family members to utilize the facility. Additionally, our experience suggests that more direct contact with our participants leads to higher retention. In addition to the direct contact during exercise sessions and the planned telephone contacts, we will offer quarterly social events such as lunches or breakfasts to discuss the program and provide updates on our progress. Additionally, approximately every three months we will provide our subjects with small gifts and incentives such as T-shirts, cups, and water bottles to celebrate their meeting certain milestones.

Training Plan for YMCA Exercise Instructors: Patricia Kluding, PhD and Eric Vidoni, PhD (postdoctoral fellow), physical therapists with experience performing exercise interventions in the stroke population, will lead our exercise intervention team. Prior to beginning the trial and periodically thereafter, Drs. Kluding and Vidoni will hold an informational training meeting at the KUMC campus with exercise instructors. This meeting will

provide an overview and rationale to the study and discuss the treatment protocols. A second training session will occur on-site at the local YMCA one week prior to beginning the initial aerobic or non-aerobic exercise visit. During these training sessions, either Dr. Kluding or Vidoni will travel to the YMCA and meet individually with the exercise instructors to ensure adequate training, compliance with the training plan, and quality data collection. Additionally, Drs. Kluding and Vidoni will be available by phone during exercise interventions to answer questions or address any unforeseen problem. While the YMCA has assured us the turnover of exercise instructors is very low, we anticipate some turnover of YMCA exercise instructors and will address this issue by ensuring that the new YMCA trainers complete the training protocol prior to initiating the intervention.

***Quality Assurance for YMCA Training and Data Collection**:* While a YMCA site is active, a member of the exercise intervention team (Kluding, Vidoni, or a research assistant) will make YMCA site visits every other week to collect data forms, review quality of data collection (e.g., completeness and accuracy of data entry) and ensure competency in the standard application of the exercise protocol through observations of the YMCA exercise instructors. If omissions or any other procedural discrepancies are identified, these will be addressed at that time with the instructors.

Nonaerobic Control Group

Participants randomized to the nonexercise control group will be asked to maintain their current level of physical activity during the 26-week active study period. They will perform outcome assessments and receive the same telephone checks as the exercise group at baseline, 26 and 52 weeks. The purpose of including a non-exercise control group is to allow adequate comparisons with the low, medium and high exercise dose groups on changes in cognitive and physiologic outcome measures. It is important to account for practice effect on cognitive testing results through a control group. Additionally, it is possible that the act of enrolling in a study for exercise may influence lifestyle changes in diet, physical activity, and health maintenance that can only be accounted by including a control group. While the effect of socialization and attention provided by a three to four day a week exercise program can only be accounted for with an active control group (i.e., stretching and toning, nonaerobic exercise classes), the primary purpose of the study as a dose-response and feasibility study does not justify the added burden and cost of adding such a group.

Study Locations

Figure 3: YMCA Locations across the Kansas City Metropolitan Area

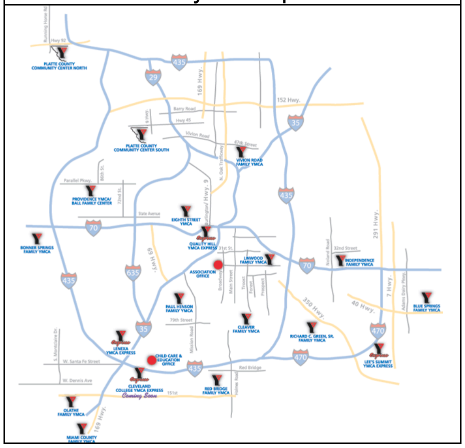

YMCA Partnership: The YMCA routinely collaborates with community organizations to encourage physical activity and well-being, and collectively, the YMCAs of the United States represent the largest collaborative organization in the country.[1] Study co-investigator Dr. Donnelly has a long and successful history of collaboration with the YMCA for his ongoing research studies. He has worked with the YMCA for 8 years on two NIH-funded (DK74909 and DK63458) studies utilizing 34 sites in Kansas City and the state of Kansas.

We will utilize 4 of the 17 YMCA centers in the greater Kansas City metropolitan area (see Figure 3). Each center is staffed by trained exercise personnel who will offer supervised aerobic treadmill exercise or non-aerobic exercise sessions at each of these sites. The YMCA trainers have personal training certification through the National Commission for Certifying Agencies and have experience working with the elderly population including individuals with mild memory problems. Our experience has been that distance to the training site makes recruitment for exercise programs difficult; thus, utilizing the community-based YMCA centers across the Kansas City greater metropolitan area will increase our base of recruitable subjects by providing easier access and shorter commutes for the subjects. Additionally, the national YMCA network is potentially an ideal platform for a multi-site trial. Thus, our local experience will importantly contribute to the design of a future definitive, multisite trial.

YMCA Safety – Special care will be taken to ensure our participants' safety. One trainer will supervise a maximum of four participants at one time. However if the participant is deemed a fall risk, the YMCA trainers are prepared to provide 1:1 supervisor to participant supervision. All of the treadmills are equipped with handrails and emergency stop switches. All of the trainers are ACLS certified and are trained in the use of portable defibrillators which are available at all of the facilities. An emergency phone to a local Emergency Medical Service is available on site.

General Clinical Research Center (GCRC): The clinical evaluations, blood draws and laboratory processing, DEXA scanning, and maximal exercise testing for $VO_2{}^{peak}$ will all be supported and conducted in collaboration with the GCRC. KUMC opened a new GCRC in December 2004 directed by Dr. Richard Barohn, M.D., the chairman of the Department of Neurology, which was later funded by the NIH in 2006. Dr. Burns serves as the Assistant Director to the GCRC and has relied heavily on the GCRC for conducting his research and has utilized the laboratory and Exercise Physiology Core extensively.

The GCRC has more than 5,000 square feet of space available for investigator-initiated clinical research. The GCRC Exercise Physiology Core is directed by Patricia Kluding, PhD (co-investigator overseeing the exercise interventions) and contains two metabolic carts (ParvoMedics TrueOne 2400), treadmill, bicycle ergometer, and electrocardiogram in addition to a Dual Energy X-ray Absorptiometry (Lunar Prodigy, version 11.2068, Madison, WI) for determining body composition. The Exercise Physiology Core is staffed by two full-time exercise physiologist. The staff and resources of the GCRC significantly defrays the cost of the laboratory and exercise testing and will greatly facilitate the conduct of the study.

Study / Outcome Measures

Aim 1: Cognitive Outcome Measures

Based on target goals of the RFA and a review of the literature on aerobic exercise interventions for cognition in healthy older adults, we selected a battery of cognitive tests that suggest a positive and differential impact of aerobic exercise across 4 cognitive domains **(TABLE 3)**: Auditory Verbal Memory, Visuospatial Memory, Set Maintenance and Switching, and Reasoning. Using CFA, as outlined below and in the preliminary data, we will generate our primary and secondary cognitive factor scores corresponding to the cognitive domains of interest in the RFA.

- **Primary Cognitive Outcome Measure: General Cognition**
- **Secondary Outcome Measures**
  - **Auditory Verbal Memory**
  - **Visual Memory**
  - **Set Maintenance and Switching**
  - **Reasoning**

We predict that general cognition will be enhanced by the aerobic exercise intervention. Greater cognitive gains will be made by individuals with more fitness gains. Finally, the intervention will affect all cognitive domains but it will affect executive functioning domains relatively more than other domains.

Test Selection: Tests selected consistently yield moderate to large sized exercise-related effects across many studies and possess good ecological validity when aggregated by cognitive domain. **Verbal Memory** translates into recall of lists, names, events, and dates. **Visual Memory** translates into the recall of faces, location, direction, and position. Although the ecological validity of **Set Maintenance and Switching** is somewhat opaque, its influence on functional performance has become apparent in the research literature (cf. Royall [124]). Studies using Verbal Fluency, Wisconsin Card Sort,[125] Trailmaking Test B,[125, 126] and Stroop [127] measures have all shown strong correlation with ADL's and IADL's. Thus, Set Maintenance and Switching appears critical to improve daily function because it facilitates *problem solving* of minor set backs encountered during a complex sequence of goal directed behavior. It is a reflection of an individual's mental flexibility and facilitates the achievement of daily care goals by compensating for impediments encountered during functional routines while maintaining the end goal in mind. We expect that this domain will yield distant transfer to daily function. Finally, **Reasoning** is a target domain of this clinical trial. Not only has several aerobic exercise interventions demonstrated a positive impact on performance[128-132] but also Reasoning has clear ecological validity. The interpretation and integration of information to reach a decision can translate into a wide range of pragmatic skills varying from complex problem solving to the comprehension of simple instructions.

Many subtests identified in our review shared common content, and administration procedures. Thus, effects sizes reported in **Table 3** aggregate across analogous subtests. For example, we collapsed across prose recall tasks of similar length and difficulty, multiple but slight variations on category and letter fluency, and list learning tasks of similar length. Preference in the selection process was given to subtests from the NACC Unified Data Set (UDS) cognitive battery[133] and the ACTIVE trial. This enhances generalizability of findings from the proposed battery across NIA program projects, as well as comparing cognitive enhancement due the proposed aerobic exercise intervention versus the cognitive training intervention from the ACTIVE trial.[134] Finally tests were selected based on their wide spread use and availability of normative data for cognitively healthy geriatric population.

This 90-minute battery will be administered to all participants at baseline, 26-week, and 52-week follow-ups. Psychometricians will not be informed of the results of the clinical evaluation or the exercise regimen to which the participants are randomized.

Table 3: Cognitive Test Battery and Reported Effect Sizes of Exercise

	Description	Effect Size Range
**Auditory Verbal Memory**		
Hopkins Verbal Learning Test †	List learning of words from semantic categories.	
Selective Reminding Test [135]	Picture list learning with structured category-cued reminding and uncued recall .	**0.14 to 0.22** [136, 137]
Logical Memory †* [138]	Prose recall of short narrative passages.	**0.76 to 1.21** [129, 139-144]
Boston Naming Test * [145]	Naming of figural line drawings.	
**Visual Memory**		
Spatial Span	Visuospatial construction using blocks to match target models.	**0.17** [146]
Visual Reproduction [138]	Memory for geometric figures.	**0.40 to 1.45** [136, 139, 141, 144, 147, 148]
Faces [138]	Memory for faces.	**0.42 to 1.12** [149]
**Set Maintenance & Switching**		
Fluency Tasks<br>Letter Fluency [150]<br>Category Fluency [151]*	Spoken wordlist generation to a target category or letter.	**0.06 to 3.67** [130, 136, 137, 147, 152, 153]
Stroop Color-Word Test	Word reading and color naming requiring set maintenance and inhibition of irrelevant information.	**.82 to 5.41** [136, 137, 141, 147, 154, 155]
Wisconsin Card Sorting Test [156]	Card-sort requiring concept formation, set maintenance, and switching.	**1.60 to 2.88** [128, 130, 137]
Trail Making Test - Part B *<br>(Trails B Minus Trails A) [157]	Line drawing between alternating numbers and letters, requiring visual search, attention, and mental flexibility.	**0.17 to 0.96** [130, 131, 141, 144, 146, 147, 152-154, 158]
Digit Span<br>(Forward & Backward)* [138]	Immediate memory for numbers, requiring information to be maintained and manipulated in mind.	**0.21 to 2.91** [131, 132, 136, 139, 141-144, 146, 147, 149, 155, 158-161]
Digit Symbol * [162]	Test of psychomotor speed involving sustained attention and transcription of digit-symbol pairs.	**0.24 to 1.45** [130, 137, 140-144, 147, 148, 152-155, 158, 159]
**Reasoning**		
Inductive Reasoning † [150]<br>Letter Series<br>Word Series<br>Number Series	Inference making based on principles derived from informative models containing letters, words, and numbers.	
Matrix Reasoning [162]	Inference making based on principles derived from informative models containing colored patterns.	**0.21 to 2.27** [128-131]
Picture Arrangement [162]	Card-sort requiring temporal sequencing and social reasoning.	**0.19** [132]

NOTE: All citations listed by number are not contained in any of the prior reviews listed
* UDS Battery Subtest
† ACTIVE Battery Subtest (or close analogue)

Cognitive Outcomes – Factor Scores: Cognitive domains listed in the neuropsychological battery are face valid aggregates of related subtests that have generated moderate to large effect sizes across several reviews on cognition and aerobic exercise. One review[163] suggests that effects sizes of aerobic exercise on executive function will be about 0.7 and for visuospatial memory about 0.4. In our review of the literature we find effect sizes for Auditory Verbal Memory range from 0.2 to 1.5.

We propose to use **Confirmatory Factor Analysis (CFA)** on baseline cognitive scores to (1) determine the best fitting empirical model of these data and (2) create factor score weights that will be applied across the 3 times of measurement in this study. CFA is a common method used to aggregate true score (common)

variance across multiple subtests and attenuate error. Thus, **factor scores** yield more reliable estimates of cognitive ability than individual tests because they are purer indices of true ability (see preliminary data).

Cognitive performance in older adults is highly variable and indices such as factor scores that aggregate true score and minimize error variance yield better indices of older adult cognitive performance.[108, 109] We have shown in previous work[107] that cognitive structure (as modeled by broad neuropsychological batteries) required a hierarchical model of both global and specific content. Our hybrid model fit both a wide range of scores from both cognitively impaired and unimpaired older adults. Further, our hybrid model combination of general and specific factors is consistent with findings that intellectual ability can best be described both as a general score and by specific mental abilities.[110] The added complexity of the hybrid model accounted for more variance in the data and left less unexplained variance in the residual (see preliminary data).

Indeed, the relatively poorer performance of the ACTIVE trial interventions[134] may be due to inadequate empirical model of cognitive performance in older adults. The ACTIVE trial used composite scores of cognitive performance (simple average of domain specific scale scores). While this method is a common sense approach to modeling domain specific variance, it is psychometrically flawed as composite scores aggregate true score variance, but also aggregate error variance. If true score variance is a relatively small component of performance, than error variance grows disproportionately large when summing across scale scores.

The current test battery has been optimized (*a priori*) to measure auditory (Verbal) memory, and executive function (Set Maintenance and Switching, and Reasoning). CFA results from the proposed trial will be based on select subtests that have been shown repeatedly in the literature to improve with aerobic fitness. Thus, the proposed factor score outcomes are expected to yield highly sensitive and reliable indices of cognitive change due to aerobic exercise intervention.

Aim 2: Peak Oxygen Consumption

Aerobic Fitness: Peak oxygen consumption (VO_2^{peak}): VO_2^{peak} is the standard, quantitative measure of aerobic fitness. Aerobic fitness is closely related to physical activity level,[116] and may be stronger than self-reported physical activity as a predictor of many health outcomes.[164] VO_2^{peak} will be assessed at baseline and 26-weeks to assess the participant's physiologic response to the exercise program. Additionally, for those randomized to the aerobic exercise group, the baseline VO_2^{peak} assessment will provide important information (i.e., maximum heart rate) to guide the exercise prescription. We have extensive experience with performing these assessments in older adults with and without cognitive impairment.[61, 95]

VO_2^{peak} is measured during a symptom-limited graded treadmill in the presence of an Advanced Cardiac Life Support (ACLS)-certified clinician (Dr. Anderson) in the GCRC. Subjects are attached to a 12-lead electrocardiograph to monitor aerobic stability and wear a non-rebreathing facemask. Heart rates are recorded at one-minute intervals and blood pressures are recorded during the last 30 seconds of each 2-minute stage. Expired air is measured for oxygen and carbon dioxide at 15-second intervals using a Parvomedics system. Gas calibrations are performed on the metabolic cart before each test according to the specifications of the manufacturer. Subjects begin walking at a pace of 1.7 miles per hour at 0% grade. Every two minutes the speed or grade is increased according to protocol (Modified Bruce protocol).[93] American College of Sports Medicine (ACSM) guidelines[116] are used to determine whether the exercise test should be terminated early and include ST-segment depression more than 2mm, increasing nervous system symptoms (i.e., ataxia, dizziness), sustained ventricular tachycardia, and chest discomfort. VO_2^{peak} is considered the highest observed value during the test.[165, 166] We will also assess whether maximal effort is achieved, as defined by achieving 3 of the 4 following criteria: a plateau in oxygen consumption with an increase in exercise intensity, a respiratory exchange ratio (RER) ≥1.0, a maximal heart rate within 90% age-predicted maximum, or volitional fatigue.[167]

Aim 3: Physical Function and ADLs

We have designed an efficient battery of functional assessments in accord with the Nagi model describing the pathway from disease to disability.[82, 168] In this model, physical impairments lead to functional limitations and eventually disability. We are assessing various measures along this continuum. We have selected as our primary functional outcome the PPT as it objectively assesses physical function by simulating everyday functional activities and has been used successfully in exercise trials,[15] including our own studies.[61, 95] The effect of exercise on measures of strength, muscle mass, gait and balance, and in turn, how these basic "building blocks" of function influence physical function and ADLs will be examined. Measures of ADLs and disability are limited in power[82] and larger, more definitive studies will be necessary to definitively assess the role of exercise in promoting functional independence. Nevertheless, we will explore two important outcome

measures of basic and instrumental ADLs and examine which performance measures are more strongly correlated with improvements in ADL tests.

Physical Performance Test: [169] The PPT provides a short, objective battery of timed physical tasks necessary for daily self-care that serves as a composite measure of physical performance. The modified PPT includes 1) writing a sentence, 2) simulated eating, 3) lifting a book and placing it on a shelf above shoulder height, 4) putting on and removing a jacket, 5) picking up a penny from the floor, 5) turning 360 degrees and 7) walking 50 feet, 8) chair rises and 9) a progressive Romberg test. Importantly, both mobility and balance components of frailty are assessed within the PPT.

Timed Up-and-Go:[170] The TUG is a valid and reliable measure of functional mobility in older adults. It correlates well with measures of balance and gait speed. Participants will be timed while rising from a chair walking 3 meters, returning and sitting.

Strength: Grip strength is a common measure in literature on aging that is associated with functional independence[171]. We will assess grip strength in both hands using a grip dynamometer as an average of three maximal grips. Lower extremity strength will be assessed via forceful knee extension with a hand held dynamomenter (MicroFET, Hoggan Ind.). The average of three measures will quantify lower extremity strength.

Body Composition: Participants will be evaluated with dual energy x-ray absorptiometry (DEXA, Lunar Prodigy, version 11.2068, Madison, WI) to determine fat-free mass, fat mass, and percent body fat at baseline, 26 weeks, and 52 weeks. DEXA uses very low X-ray doses (0.02mREM) corresponding to less than several hours of background exposure, and is able to detect changes in body composition on the order of 1.6-3.8%.[172, 173] In addition to DEXA, body composition is assessed with standard anthropometric measures. Body mass is determined using a digital scale accurate to ±0.1 kg (Seca Platform Scale, model 707, Seca Corp., Columbia, MD) prior to assessment of body composition by DEXA. The subjects are weighed prior to breakfast and after attempting to void. Circumference measurements are taken at the smallest girth around the trunk and at the widest protrusion of the buttocks. Three circumference measurements are taken per site with a Gullick II circumference measurement tape. Additional measures are taken if the measurements vary by more than 2 cm. The average of the 3 measurements is used in the statistical analysis.

Basic and Instrumental Activities of Daily Living: The Barthel Index will be used to measure independence in basic daily activities. The Barthel Index is a reliable measure of performance on independence in ADLs, such as dressing, toileting and feeding.[174, 175] We will assess functional improvements from exercise participation using the Alzheimer's Disease Cooperative Study-ADL scale.[176] This is an IADL living inventory developed by the ADCS to assess functional performance of patients with AD. The inventory is given to the study partner regarding the subject's activities during the preceding four weeks. Questions of complex planning such as community travel and participation distinguish this survey from the Barthel Index and PPT.

Covariates / Other Outcome Measures

Adherence Rates: The primary measure of adherence will be percent of expended calories per week divided by the prescribed calories per week over the 26-week intervention. We will also assess adherence as number of sessions attended.

Adverse Events: Adverse events will be monitored and tracked over the study with a daily exercise symptom checklist and monitoring of new medical co-morbidities. Of particular interest are exercise-limiting injuries, adverse events resulting in withdrawal from the study, and falls (categorized as injurious vs. non-injurious).

Community Healthy Activities Model Program for Seniors (CHAMPS): The CHAMPS questionnaire will be administered to the caregiver at baseline, 26-weeks, and 52-weeks to assess levels of physical activity in both groups. CHAMPS is a 41-item measure that was developed to assess levels of physical activity in older adults. CHAMPS is a robust measure (unaffected by seasonal bias) designed to capture a wide variety of physical activities in older adults. The instrument includes typical exercises and recreational activities as well as routine physical activities performed throughout the course of a typical day in older adults. This instrument has been shown to be a valid and reliable method to assess energy expenditures in older adults over the course of 6-month[177] and 12-month[178, 179] interventions. The CHAMPS evaluation will allow us to assess both groups for changes in physical activity, including the possibility that the control group will adopt healthier lifestyles and potentially confound our results.

SF-36 Quality of Life Questionnaire:[180] The **SF-36** is a well-recognized, easily administered survey which measures eight domains of health including physical functioning, role limitations due to physical health, bodily pain, general health perceptions, vitality, social functioning, role limitations due to emotional problems, and

mental health. We will use the SF-36 primarily to assess Quality of Life associated with the exercise intervention.

Geriatric Depression Scale: We will continue to assess depressive symptoms at two year follow up with the Geriatric Depression Scale[181]administered to the collateral source. Individuals with major depression are excluded at baseline although depressive symptoms may emerge during follow up as individuals with AD are at high risk of depression. Depression has been shown to influence CR fitness in nondemented women[182] and is therefore a possible confounding factor in the assessment of CR fitness in AD.

Statistical Analyses

This pilot study is intended to inform and advance the design of a subsequent clinical trial and will allow effect size estimates for powering a definitive study, consistent with the objectives of the RFA.

The **sample size** of 100 was determined based on reported exercise-related effect sizes on cognition (d=0.40)[2] and a conservative estimate of 20% drop-out. To test our *a priori* hypothesis that exercise will have a dose-response relationship with cognition (assuming a power of 80% and a 1-tailed alpha = 0.05), at least 68 subjects are necessary (SAS, PROC POWER). We will slightly over enroll to account for effect sizes less than expected and to ensure reliable cognitive factor scores. This sample size will also provide greater than 80% power to detect linear, quadratic and cubic trends for aerobic fitness (d=0.35)[88] and physical function (PPT, d=1.75)[15] across the four groups (1-tailed alpha = 0.05). The RFA indicates the pilot study does not need adequate power to detect the effect size on cognition of the intervention. Nevertheless, the current study design provides adequate power (80% with a one-tailed alpha = 0.05) using ANOVA to detect an effect size greater than 0.32 of exercise on cognition across the three dose groups vs. control.

Aim 1: Examine the dose-response of aerobic exercise on cognitive function in individuals 75 years and older in a 26-week, community-based, supervised exercise program.

We predict that general cognition will be enhanced by the aerobic exercise intervention. Greater cognitive gains will be made by individuals with more fitness gains. Finally, the intervention will affect all cognitive domains but it will affect the executive functioning domain (Set Maintenance and Switching) relatively more than other domains.

The primary analytic plan for aim 1 consists of four phases:

1. Baseline CFA and computation of factor score weights
2. Dose-response analysis of the effect of various doses on cognition (primary outcome General Factor score; secondary outcomes of four cognitive domains: Auditory Verbal Memory, Visual Memory, Set Maintenance and Switching, and Reasoning).
3. Assessment of adherence across dose groups.
4. Durability analyses of the changes in cognition over the 52 weeks of the study.

Baseline CFA and computation of factor score weights: Cross-sectional cognitive subtest scores from all participants at the baseline time of measurement (N=100) will be used in a multistep CFA (MPlus, version 5). First the relative fit of four candidate hypothetical models (**Figure 1**) will examine domain content, factor loadings, and factor interrelationships. Model A hypothesizes a single general factor. Model B tests three independent factors. Model C allows the three factors to be correlated. A hybrid Model D allows both three specific factors and a general first-order factor. The empirical validity of each model (i.e., how well it fit the observed data) will be assessed using goodness-of-fit indices.[183] Model selection is primarily based on differences in the root mean square error of approximation (RMSEA[184]), which is a measure of discrepancy between predicted and observed model values; values closer to 0 indicate better fit (preferred values < .09). In accordance with more recent guidelines, better fitting models will be accepted when the change in the RMSEA (ΔRMSEA) is greater than 0.02.[185] In the event that RMSEA values are subthreshold, standard -2 Log Likelihood ratio tests will be conducted. The best fitting and simplest model out of the 4 possible will be used to generate factor score weights. Factor score weighting will be applied to the follow up assessments.

There is disagreement about what constitutes sufficient sample size for CFA, especially in clinical research where data collection is intensive and sample size often limited. Although larger sample sizes than those presented here are commonly used, recent Monte Carlo modeling work[32] indicates that sample size is conditional on data and model characteristics. Good-fitting models can be identified with relatively smaller sample sizes (N=100) when reliable measures are used. The meta-analytic literature insures that true score variance in the measures used here is moderate to large and reliably consistent across many studies. Given that this RFA is exploratory in nature and seeks proof of principle, the baseline sample size of 100 will provide adequate power to conduct this CFA model fitting procedure. We have conducted the same analyses using a

sample of 71 nondemented PD individuals and attained good fit (RMSEA = .04; **Figure 1**). The cognitive effect sizes estimated in this study will not differ greatly from what would be generated in a large scale clinical trial. Further, the use of CFA economizes the true score variance of cognitive and physical function thereby making them more reliable estimates than individual subtests.

Exercise Dose-Response (Trend) Analysis for Cognition: Our *a priori* hypothesis is that exercise will have a dose-response relationship with general cognition (primary cognitive outcome measure) and across cognitive domains (secondary outcome measures of Auditory Verbal Memory, Visual Memory, Set Maintenance and Switching, and Reasoning). However, we expect the greatest change to occur in Set Maintenance and Switching. Because the intervention design is a linear prescription similar to the DREW study[88] we expect a simple linear trend of increasing exercise-related cognitive benefits across groups; however, it is also possible that increasing exercise regimens will have alternate effects in this elderly population and alternate trends are possible. For example, the 50% regimen may not return significant $VO2^{peak}$ increases while the 150% may have diminishing returns (perhaps related to decreased adherence or adverse effects) and result a cubic trend.

Polynomial contrasts using ANOVA (SAS; PROC GLM) will assess for linear, quadratic, and cubic trends of cognitive changes in the primary and secondary cognitive outcome measures. ANOVA with group contrasts will also assess group differences in cognitive performance across the four dose groups. If skewness or heterogeneity of variance exists among any of the outcomes, tests will be conducted with bootstrap resampling techniques (SAS; PROC MULTTEST). This technique approximates a non-parametric distribution of minimum p-value given all possible tests and final p-values are adjusted accordingly.

We will also test if important covariates (i.e., education, gender, depressive symptoms, comorbidities) are related to cognition or exercise-related cognitive change. We will adopt a covariate into an analysis if it correlates significantly with an outcome both at baseline and over time. If the distribution of any potential covariate is skewed even with variable transformation, it will be tested with the Kruskal-Wallis one way analysis of variance (PROC NPAR1WAY).

Adherence: To test whether increasing exercise doses impact adherence, ANOVA will be used to compare the primary measure of adherence (average expended calories per week divided by the prescribed calories per week over the 26-week study) across the 3 exercise groups. Using ANCOVA, we will also examine if additional factors are related to adherence such as age, gender, co-morbidities, injuries, and distance lived from the facility.

Durability of Exercise Related Cognitive Changes over 52-weeks: To assess if exercise-related cognitive changes persist after completion of the 26-week exercise intervention, we will use piecewise linear regression to examine cognitive performance across the entire study period (baseline, 26-week, and 52-week evaluations). The piecewise change model will specify a point of inflection at 26-weeks (post-intervention follow-up) and test for group differences due to the intervention as well as group-wise rates of change (Group X Time) in the pre and post-intervention periods (SAS; PROC MIXED). Resultant test statistics represent a simultaneous comparison of gains in cognitive ability due to the intervention and subsequent change due to its cessation. Comparison of the rates of gains versus the rates of loss addresses this RFA target goal of characterizing the durability of the changes made in cognition due to the exercise intervention. An example of this regression is provided in the Preliminary Section for cognitive data, Figure 2. We have used this design to investigate weight loss associated with AD[186], cognitive change due to dementia (see above), and it is widely applied in epidemiological research.[111] We will also use similar analytic technique to assess if physical activity levels (estimated using the CHAMPS) are maintained or decline after completion of the intervention across the four groups.

Aims 2 and 3: Examine the dose-response of exercise on aerobic fitness (aim 2) and physical function (aim 3).

Change in the primary measures of aerobic fitness ($VO2^{peak}$) and physical function (PPT) between baseline and 26-week follow-up) will be assessed across the control and 3 exercise intervention groups with an ANOVA omnibus test (SAS; PROC GLM) using analogous techniques assessing the dose-response for cognitive outcomes. Secondary analyses will assess change in physiologic measures of function including lean mass, strength, and gait / balance (timed up-and-go). The relationship of potential covariates (adherence, age, gender, co-morbidities) with exercise-related changes in outcomes for aims 2 and 3 will be assessed using similar analyses as described above.

Additional Analyses of Interest

Mediation analyses will be conducted to investigate the relationship between exercise dose (X; average expended calories per week), fitness (M; % gain in VO_2^{peak}), and cognition (Y; change in general factor score from baseline to 26-weeks). A bootstrap re-sampling technique[187] will be used to assess possible standard series of causal relationships: 1) the relationship between exercise dose and cognition 2) the relationship between fitness and cognition, and 3) the relationship between exercise, fitness, and cognition. This will yield an estimate of the amount of mediation that fitness plays in the relationship between exercise and cognition to examine the ***aerobic fitness hypothesis***.

Assessment of Optimal Exercise Dose: A goal of this pilot study is to determine an optimal exercise dose in older adults that maximizes gains in cognition and aerobic fitness. We will conduct a series of exploratory analyses to examine which dose provides the most benefit to both cognition and aerobic fitness. Receiver Operator Characteristic (ROC) curves will be generated assessing fitness and cognitive gains (% change $VO2^{peak}$ x % change in general cognitive factor score). The area under the curves for each dose will be compared[188] across the three doses to inform the choice of an optimal exercise dose for future studies.

Data management

Clinical Data Management System: The KU Alzheimer and Memory Program is currently transitioning to an electronic database system, the Comprehensive Research Information System (CRIS). This secure web-based system utilizes the Velos eResearch system and allows data and protocol information to be entered efficiently and in a standardize format compliant with NIH reporting standards. The system is ideal for supporting multi-site trials and importantly the proposed pilot trial will give us the opportunity to fully refine the forms and design a robust data management system necessary to support a multi-site initiative.

CRIS is a web-based software application that includes capabilities to: 1) create and edit participant data, such as demographics, labs, medications, etc.;2) track the development of study protocols, amendments, and IRB approvals/renewals; 3) create screening and enrolling criteria; 4) create and disseminate case report forms; 5) create and maintain sample banks and associate samples with clinical data; 6) create participant schedules and record clinical results and participant status in research protocols; 7) create user and multi-organization research networks; 8) record, maintain, and report adverse events; 9) store and report on all participant- and study-level clinical data; 10) conduct study queries and generate reports; 12) export data to SAS and Excel. CRIS is also HL-7 compliant and configured to easily integrate with internal as well as third-party lab systems or electronic medical records. The system includes programming to maintain compliance with CFR Part 11 and other industry and federal standards. The system can support multi-center, cooperative group and investigator-initiated research through advanced technology and security features.

Data Collection Forms: All data will be collected on standard source documents followed by entry into standard case report forms in the CRIS system. We have developed a preliminary set of source forms and case report forms through our 12-week pilot study. These forms include daily exercise data forms used by the YMCA exercise instructors and source documents for outcome and clinical assessments. Initial development will occur in a test environment of CRIS where forms will be reviewed for completeness and accuracy. Mock data collection forms will be completed to ensure compatibility with the data being collected in the source documentation and to validate the range, logic and other edit checks in the web-based data entry system. The Center for Biostatistics and Advanced Informatics ensures data security by managing all data on a secure server that has role-based access that is password protected. All files that are modified are backed up daily, with complete backups of the server on a weekly basis. All data are stored in a HIPAA compliant manner.

Adverse Events

Adverse events are defined as any untoward medical occurrence in study participants or others, which does not necessarily have to be a causal relationship with the study treatment. The seriousness of the adverse event will be determined using the National Cancer Institute Common Terminology Criteria for Adverse Events (CTCAE) v3.0,[95] and any adverse event rated category 2 or greater is considered serious.

All maximal exercise testing will be monitored by a physician and if any clinically significant ECG changes are identified, we will contact the participant's primary care physician, with the participant's consent, and assist in referring the participant for cardiology follow-up. Adverse events will be assessed at every visit as well as during telephone assessments every 6 weeks. If the participant or exercise instructor reports adverse events or complaints, the participant will be evaluated by the unblinded investigator (Dr. Burns). The unblinded safety investigator will have access to training logs, telephone assessment forms, and any study-related results. Incidental or new findings of clinical concern will first be discussed with the subject and then communicated to the subject's primary care physician, if consent to do so is provided.

References

1. Kramer AF, Hahn S, Cohen NJ et al. Ageing, fitness and neurocognitive function. Nature. 1999;400:418-419
2. Colcombe S, Kramer AF. Fitness effects on the cognitive function of older adults: A meta-analytic study. Psychological Science. 2003;14:125-130
3. Friedland RP, Fritsch T, Smyth KA et al. Midlife recreational activity inversely associated with Alzheimer's disease risk. Proc Natl Acad Sci. 2001;98:3440-3445
4. Laurin D, Verreault R, Lindsay J et al. Physical activity and risk of cognitive impairment and dementia in elderly persons. Arch Neurol. 2001;58:498-504
5. Yaffe K, Barnes D, Nevitt M et al. A Prospective Study of Physical Activity and Cognitive Decline in Elderly Women: Women Who Walk. Archives of Internal Medicine. 2001;161:1703-1708
6. Pignatti F, Rozzini R, Trabucchi M, Yaffe K. Physical Activity and Cognitive Decline in Elderly Persons. Archives of Internal Medicine. 2002;162:361-362
7. Dustman RE, Ruhling RO, Russell EM et al. Aerobic Exercise Training and Improved Neuropsychological Function of Older Individuals. Neurobiology of Aging. 1984;5:35-42
8. Hassmen P, Koivula N. Mood, physical working capacity and cognitive performance in the elderly as related to physical activity. Aging-Clinical and Experimental Research. 1997;9:136-142
9. Williams P, Lord SR. Effects of group exercise on cognitive functioning and mood in older women. Australian and New Zealand Journal of Public Health. 1997;21:45-52
10. Hill RD, Storandt M, Malley M. The impact of long-term exercise training on psychological function in older adults. J Gerontol. 1993;48:12-17
11. Barnes DE, Yaffe K, Satariano WA, Tager IB. A Longitudinal Study of Cardiorespiratory Fitness and Cognitive Function in Healthy Older Adults. Journal of the American Geriatrics Society. 2003;51:459-465
12. Weuve J, Kang JH, Manson JE et al. Physical Activity, Including Walking, and Cognitive Function in Older Women. JAMA: The Journal of the American Medical Association. 2004;292:1454-1461
13. Abbott RD, White LR, Ross GW et al. Walking and Dementia in Physically Capable Elderly Men. JAMA: The Journal of the American Medical Association. 2004;292:1447-1453
14. Nelson ME, Rejeski WJ, Blair SN et al. Physical activity and public health in older adults: recommendation from the American College of Sports Medicine and the American Heart Association. Med Sci Sports Exerc. 2007;39:1435-1445
15. Villareal DT, Banks M, Sinacore DR et al. Effect of Weight Loss and Exercise on Frailty in Obese Older Adults. Arch Intern Med. 2006;166:860-866
16. Gu MO, Conn VS. Meta-analysis of the effects of exercise interventions on functional status in older adults. Res Nurs Health. 2008
17. Statistics FIFoA-R. Older Americans 2008: Key Indicators of Well-Being. In: Stastics FAFoA-R, ed. Washington, DC, 2009
18. Bureau USC. Population Projections, 2008
19. Aging NIo. Growing Older in America: the Healthy and Retirement Study. In: Karp F, ed. Bethesda, MD: National Institutes of Health, 2007
20. Suthers K, Kim JK, Crimmins E. Life expectancy with cognitive impairment in the older population of the United States. J Gerontol B Psychol Sci Soc Sci. 2003;58:S179-186
21. Freedman VA, Schoeni RF, Martin LG, Cornman JC. Chronic Conditions and the Decline in Late-Life Disability. TRENDS Working Paper 06-05, 2006
22. Prigerson HG. Costs to Society of Family Caregiving for Patients with End-Stage Alzheimer's Disease. The New England Journal of Medicine. 2003;349:1891-1892
23. Kawas CH, Katzman, R. Epidemiology of dementia and Alzheimer disease. In: Katzman R, Bick, K. L., Sisodia, S. S., ed. Alzheimer Disease. 2nd ed. Philidelphia, PA Lippincot Williams & Wilkins, 1999:95-116.
24. Keysor JJ, Jette AM. Have we oversold the benefit of late-life exercise? J Gerontol A Biol Sci Med Sci. 2001;56:M412-423
25. Prevalence of no leisure-time physical activity--35 States and the District of Columbia, 1988-2002. MMWR Morb Mortal Wkly Rep. 2004;53:82-86
26. Prevention CfDCa. US Physical Activity Statistics: Department of Health and Human Services, 2008

27. Blair SN, Church TS. The Fitness, Obesity, and Health Equation: Is Physical Activity the Common Denominator? JAMA: The Journal of the American Medical Association. 2004;292:1232-1234
28. Blair SN, Kampert JB, Kohl HW, III et al. Influences of cardiorespiratory fitness and other precursors on cardiovascular disease and all-cause mortality in men and women. JAMA: The Journal of the American Medical Association. 1996;276:205-210
29. Lakka TA, Laukkanen JA, Rauramaa R et al. Cardiorespiratory Fitness and the Progression of Carotid Atherosclerosis in Middle-Aged Men. Annals of Internal Medicine. 2001;134:12-20
30. Kurl S, Laukkanen JA, Rauramaa R et al. Cardiorespiratory Fitness and the Risk for Stroke in Men. Archives of Internal Medicine. 2003;163:1682-1688
31. Sandvik L, Erikssen J, Thaulow E et al. Physical Fitness as a Predictor of Mortality among Healthy, Middle-Aged Norwegian Men. The New England Journal of Medicine. 1993;328:533-537
32. Neeper SA, Gomezpinilla F, Choi J, Cotman C. Exercise and Brain Neurotrophins. Nature. 1995;373:109-109
33. Churchill JD, Galvez R, Colcombe S et al. Exercise, experience and the aging brain*1. Neurobiology of Aging. 2002;23:941-955
34. van Praag H, Christie BR, Sejnowski TJ, Gage FH. Running enhances neurogenesis, learning, and long-term potentiation in mice. Proceedings of the National Academy of Sciences of the United States of America. 1999;96:13427-13431
35. Barde YA. Neurotrophins: a family of proteins supporting the survival of neurons. Prog.Clin Biol Res. 1994;390:45-56
36. Stummer W, Weber K, Tranmer B et al. Reduced mortality and brain damage after locomotor activity in gerbil forebrain ischemia. Stroke; a Journal Of Cerebral Circulation. 1994;25:1862-1869
37. Carro E, Trejo JL, Busiguina S, Torres-Aleman I. Circulating insulin-like growth factor I mediates the protective effects of physical exercise against brain insults of different etiology and anatomy. The Journal Of Neuroscience: The Official Journal Of The Society For Neuroscience. 2001;21:5678-5684
38. Lu B, Chow A. Neurotrophins and hippocampal synaptic transmission and plasticity. J Neurosci Res. 1999;58:76-87
39. Black JE, Isaacs KR, Anderson BJ et al. Learning causes synaptogenesis, whereas motor activity causes angiogenesis, in cerebellar cortex of adult rats. Proceedings of the National Academy of Sciences of the United States of America. 1990;87:5568-5572
40. Isaacs KR, Anderson BJ, Alcantara AA et al. Exercise and the brain: angiogenesis in the adult rat cerebellum after vigorous physical activity and motor skill learning. Journal Of Cerebral Blood Flow And Metabolism: Official Journal Of The International Society Of Cerebral Blood Flow And Metabolism. 1992;12:110-119
41. Young D, Lawlor PA, Leone P et al. Environmental enrichment inhibits spontaneous apoptosis, prevents seizures and is neuroprotective. Nature Medicine. 1999;5:448-453
42. Cotman CW, Berchtold NC. Exercise: a behavioral intervention to enhance brain health and plasticity. Trends in Neurosciences. 2002;25:295-301
43. Escorihuela RM, Tobena A, Fernandez-Teruel A. Environmental enrichment and postnatal handling prevent spatial learning deficits in aged hypoemotional (Roman high-avoidance) and hyperemotional (Roman low-avoidance) rats. Learning & Memory (Cold Spring Harbor, N.Y.);2:40-48
44. Adlard PA, Perreau VM, Pop V, Cotman CW. Voluntary Exercise Decreases Amyloid Load in a Transgenic Model of Alzheimer's Disease. Journal of Neuroscience. 2005;25:4217-4221
45. Seals DR, Hagberg JM, Hurley BF et al. Effects of endurance training on glucose tolerance and plasma lipid levels in older men and women. JAMA: The Journal of the American Medical Association. 1984;252:645-649
46. Hughes VA, Fiatarone MA, Fielding RA et al. Exercise increases muscle GLUT-4 levels and insulin action in subjects with impaired glucose tolerance. Am J Physiol. 1993;264:E855-E862
47. Kirwan JP, Kohrt WM, Wojta DM et al. Endurance exercise training reduces glucose-stimulated insulin levels in 60- to 70-year-old men and women. J Gerontol. 1993;48:M84-M90
48. Cox JH, Cortright RN, Dohm GL, Houmard JA. Effect of aging on response to exercise training in humans: skeletal muscle GLUT-4 and insulin sensitivity. Journal of Applied Physiology. 1999;86:2019-2025
49. Kahn SE, Larson VG, Beard JC et al. Effect of exercise on insulin action, glucose tolerance, and insulin secretion in aging. Am J Physiol. 1990;258:E937-E943

50. Houmard JA, Tyndall GL, Midyette JB et al. Effect of reduced training and training cessation on insulin action and muscle GLUT-4. Journal of Applied Physiology. 1996;81:1162-1168
51. Langa KM, Foster NL, Larson EB. Mixed Dementia: Emerging Concepts and Therapeutic Implications. JAMA: The Journal of the American Medical Association. 2004;292:2901-2908
52. Yaffe K, Kanaya A, Lindquist K et al. The Metabolic Syndrome, Inflammation, and Risk of Cognitive Decline. JAMA: The Journal of the American Medical Association. 2004;292:2237-2242
53. Expert Panel on Detection EaToHBCiA. Executive Summary of the Third Report of the National Cholesterol Education Program (NCEP) Expert Panel on Detection, Evaluation, and Treatment of High Blood Cholesterol in Adults (Adult Treatment Panel III). JAMA: The Journal of the American Medical Association. 2001;285:2486-2497
54. Ferreira I, Twisk JWR, van Mechelen W et al. Development of Fatness, Fitness, and Lifestyle From Adolescence to the Age of 36 Years: Determinants of the Metabolic Syndrome in Young Adults: The Amsterdam Growth and Health Longitudinal Study. Archives of Internal Medicine. 2005;165:42-48
55. Launer LJ, Masaki K, Petrovitch H et al. The association between midlife blood pressure levels and late-life cognitive function. The Honolulu-Asia Aging Study. JAMA: The Journal of the American Medical Association. 1995;274:1846-1851
56. Gustafson D, Rothenberg E, Blennow K et al. An 18-Year Follow-up of Overweight and Risk of Alzheimer Disease. Archives of Internal Medicine. 2003;163:1524-1528
57. Fujisawa Y, Sasaki K, Akiyama K. Increased insulin levels after OGTT load in peripheral blood and cerebrospinal fluid of patients with dementia of Alzheimer type. Biological Psychiatry. 1991;30:1219-1228
58. Ott A, Stolk RP, van Harskamp F et al. Diabetes mellitus and the risk of dementia: The Rotterdam Study. Neurology. 1999;53:1937
59. Peila R, Rodriguez BL, White LR, Launer LJ. Fasting insulin and incident dementia in an elderly population of Japanese-American men. Neurology. 2004;63:228-233
60. Schmidt R, Schmidt, H., Curb, J.D., Masaki, K., White, L. R., Launer, L. J. Early inflamation and dementia: A 25-year follow-up of the Honolulu-Asia Aging Study. Annals of Neurology. 2002;52:168-174
61. Burns JM, Donnelly JE, Anderson HS et al. Cardiorespiratory Fitness and Brain Atrophy in Early Alzheimer's Disease. Neurology. 2008;In Press
62. Colcombe SJ, Erickson KI, Raz N et al. Aerobic Fitness Reduces Brain Tissue Loss in Aging Humans. Journals of Gerontology Series A: Biological Sciences and Medical Sciences. 2003;58:M176-M180
63. Colcombe SJ, Erickson KI, Scalf PE et al. Aerobic exercise training increases brain volume in aging humans. J Gerontol A Biol Sci Med Sci. 2006;61:1166-1170
64. Colcombe SJ, Kramer AF, Erickson KI et al. Cardiovascular fitness, cortical plasticity, and aging. Proceedings of the National Academy of Sciences. 2004;101:3316-3321
65. Dustman RE, Emmerson RY, Ruhling RO et al. Age and fitness effects on EEG, ERPs, visual sensitivity, and cognition. Neurobiol.Aging. 1990;11:193-200
66. van Boxtel MP, Paas FG, Houx PJ et al. Aerobic capacity and cognitive performance in a cross-sectional aging study. Med Sci Sports Exerc. 1997;29:1357-1365
67. Shay KA, Roth DL. Association Between Aerobic Fitness and Visuospatial Performance in Healthy Older Adults. Psychology and Aging. 1992;7:15-24
68. Friedland RP, Fritsch T, Smyth KA et al. Patients with Alzheimer's disease have reduced activities in midlife compared with healthy control-group members. Proceedings of the National Academy of Sciences. 2001;98:3440-3445
69. Albert MS, Jones K, Savage CR et al. Predictors of cognitive change in older persons: MacArthur studies of successful aging. Psychol Aging. 1995;10:578-589
70. Larson EB, Wang L, Bowen JD et al. Exercise Is Associated with Reduced Risk for Incident Dementia among Persons 65 Years of Age and Older. Ann Intern Med. 2006;144:73-81
71. Lautenschlager NT, Cox KL, Flicker L et al. Effect of Physical Activity on Cognitive Function in Older Adults at Risk for Alzheimer Disease: A Randomized Trial. Vol. 300, 2008:1027-1037
72. Larson EB. Physical Activity for Older Adults at Risk for Alzheimer Disease. Vol. 300, 2008:1077-1079
73. Chandler JM, Hadley EC. Exercise to improve physiologic and functional performance in old age. Clin Geriatr Med. 1996;12:761-784
74. Kohrt WM, Malley MT, Coggan AR et al. Effects of gender, age, and fitness level on response of VO2max to training in 60-71 yr olds. J Appl Physiol. 1991;71:2004-2011

75. McCartney N, Hicks AL, Martin J, Webber CE. Long-term resistance training in the elderly: effects on dynamic strength, exercise capacity, muscle, and bone. J Gerontol A Biol Sci Med Sci. 1995;50:B97-104
76. McCartney N, Hicks AL, Martin J, Webber CE. A longitudinal trial of weight training in the elderly: continued improvements in year 2. J Gerontol A Biol Sci Med Sci. 1996;51:B425-433
77. Cress ME, Buchner DM, Questad KA et al. Exercise: effects on physical functional performance in independent older adults. J Gerontol A Biol Sci Med Sci. 1999;54:M242-248
78. Buchner DM, Cress ME, de Lateur BJ et al. The effect of strength and endurance training on gait, balance, fall risk, and health services use in community-living older adults. J Gerontol A Biol Sci Med Sci. 1997;52:M218-224
79. Binder EF, Schechtman KB, Ehsani AA et al. Effects of exercise training on frailty in community-dwelling older adults: results of a randomized, controlled trial. J Am Geriatr Soc. 2002;50:1921-1928
80. Evans WJ. Effects of exercise on body composition and functional capacity of the elderly. J Gerontol A Biol Sci Med Sci. 1995;50 Spec No:147-150
81. Bean JF, Vora A, Frontera WR. Benefits of exercise for community-dwelling older adults. Arch Phys Med Rehabil. 2004;85:S31-42; quiz S43-34
82. Guralnik JM, Ferrucci L. Assessing the building blocks of function: utilizing measures of functional limitation. Am J Prev Med. 2003;25:112-121
83. Kesaniemi YK, Danforth E, Jr., Jensen MD et al. Dose-response issues concerning physical activity and health: an evidence-based symposium. Med Sci Sports Exerc. 2001;33:S351-358
84. Lee IM. Dose-Response Relation Between Physical Activity and Fitness: Even a Little Is Good; More Is Better. Vol. 297, 2007:2137-2139
85. Manson JE, Greenland P, LaCroix AZ et al. Walking Compared with Vigorous Exercise for the Prevention of Cardiovascular Events in Women. The New England Journal of Medicine. 2002;347:716-725
86. Manini TM, Everhart JE, Patel KV et al. Daily Activity Energy Expenditure and Mortality Among Older Adults. Vol. 296, 2006:171-179
87. Lee IM, Rexrode KM, Cook NR et al. Physical Activity and Coronary Heart Disease in Women: Is "No Pain, No Gain" Passe? Vol. 285, 2001:1447-1454
88. Church TS, Earnest CP, Skinner JS, Blair SN. Effects of Different Doses of Physical Activity on Cardiorespiratory Fitness Among Sedentary, Overweight or Obese Postmenopausal Women With Elevated Blood Pressure: A Randomized Controlled Trial. Vol. 297, 2007:2081-2091
89. Etnier JL, Nowell PM, Landers DM, Sibley BA. A meta-regression to examine the relationship between aerobic fitness and cognitive performance. Brain Research Reviews. 2006;52:119-130
90. Angevaren M, Aufdemkampe G, Verhaar HJ et al. Physical activity and enhanced fitness to improve cognitive function in older people without known cognitive impairment. Cochrane Database Syst Rev. 2008:CD005381
91. Brookmeyer R, Gray S, Kawas C. Projections of Alzheimer's disease in the United States and the public health impact of delaying disease onset. American Journal of Public Health. 1998;88:1337-1342
92. Birks J. Cholinesterase inhibitors for Alzheimer's disease. Cochrane Database Syst Rev. 2006:CD005593
93. Hollenberg M, Ngo LH, Turner D, Tager IB. Treadmill Exercise Testing in an Epidepiologic Study of Elderly Subjects. Journal of Gerontology: Biological Sciences. 1998;53A:259-267
94. Balady GJ, Berra KA, Golding LA et al. American College of Sport Medicine's Guidelines for exercise testing and prescription Sixth ed. Baltimore: Williams & Wilkins, 2007
95. Burns JM, Mayo MS, Anderson HS et al. Cardiorespiratory Fitness in Early-Stage Alzheimer’s Disease. . Alzheimer Dis Assoc Disord. 2008;22:39-46
96. Donnelly JE, Hill JO, Jacobsen DJ et al. Effects of a 16-month randomized controlled exercise trial on body weight and composition in young, overweight men and women: the Midwest Exercise Trial. Archives of Internal Medicine. 2003;163:1343-1350
97. Donnelly JE, Hill JO, Jacobsen DJ et al. Effects of a 16-month randomized controlled exercise trial on body weight and composition in young, overweight men and women: the Midwest Exercise Trial. Arch Intern Med. 2003;163:1343-1350
98. Donnelly JE, Jacobsen DJ, Heelan KS et al. The effects of 18 months of intermittent vs. continuous exercise on aerobic capacity, body weight and composition, and metabolic fitness in previously sedentary, moderately obese females. Int J Obes.Relat Metab Disord. 2000;24:566-572

99. Donnelly JE, Kirk EP, Jacobsen DJ et al. Effects of 16 mo of verified, supervised aerobic exercise on macronutrient intake in overweight men and women: the Midwest Exercise Trial. Am J Clin Nutr. 2003;78:950-956
100. Billinger SA, Tseng BY, Kluding PM. Modified total-body recumbent stepper exercise test for assessing peak oxygen consumption in people with chronic stroke. Phys Ther. 2008;88:1188-1195
101. Kluding P, Billinger SA. Exercise-induced changes of the upper extremity in chronic stroke survivors. Top Stroke Rehabil. 2005;12:58-68
102. Kluding P, McGinnis PQ. Multidimensional exercise for people with Parkinson's disease: a case report. Physiother Theory Pract. 2006;22:153-162
103. Kluding PM, Santos M. Effects of ankle joint mobilizations in adults poststroke: a pilot study. Arch Phys Med Rehabil. 2008;89:449-456
104. Colcombe S, Kramer AF. Fitness effects on the cognitive function of older adults: a meta-analytic study. Psychol Sci. 2003;14:125-130
105. Etnier JL, Nowell PM, Landers DM, Sibley BA. A meta-regression to examine the relationship between aerobic fitness and cognitive performance. Brain Res Rev. 2006;52:119-130
106. Heyn P, Abreu BC, Ottenbacher KJ. The effects of exercise training on elderly persons with cognitive impairment and dementia: a meta-analysis. Arch Phys Med Rehabil. 2004;85:1694-1704
107. Johnson DK, Storandt M, Morris JC et al. Cognitive profiles in dementia: Alzheimer disease vs nondemented aging. Neurology. In Press
108. Salthouse TA, Ferrer-Caja E. What needs to be explained to account for age-related effects on multiple cognitive variables? Psychol Aging. 2003;18:91-110
109. Zelinski EM, Lewis KL. Adult age differences in multiple cognitive functions: differentiation, dedifferentiation, or process-specific change? Psychol Aging. 2003;18:727-745
110. Pretz JE, Sternberg RJ. Unifying the field: Cognition and intelligence, 2005:Sternberg, Robert J.; Pretz, Jean E. (2005). Cognition and intelligence: Identifying the mechanisms of the mind. (p
111. Fitzmaurice G, Laird NM, Ware JH. Applied Longitudinal Analyses. Hoboken, NJ: John Wiley & Sons, Inc., 2004
112. Galvin JE, Johnson DK, Morris JC. Cognitive profiles of Parkinson's disease and Lewy body demetias. National Institute of Aging, Alzheimer's Disease Center Directors' Meeting, 2008
113. Johnson DK, Morris JC, Galvin JE. Verbal and visuospatial deficits in dementia with Lewy bodies. Neurology. 2005;65:1232-1238
114. Johnson DK, Storandt M, Morris JC, Galvin JE. Longitudinal Study of the Transition from Healthy Aging to Alzheimer's Disease. Neurology. Submitted
115. Morss GM, Jordan AN, Skinner JS et al. Dose Response to Exercise in Women aged 45-75 yr (DREW): design and rationale. Med Sci Sports Exerc. 2004;36:336-344
116. American College of Sports M. Guidelines for exercise testing and prescription Baltimore: Williams & Wilkins, 2000
117. NIH. Physical activity and cardiovascular health. NIH Consensus Development Panel on Physical Activity and Cardiovascular Health. JAMA. 1996;276:241-246
118. Brandt J, Folstein M. The telephone interview for cognitive status. Neuropsychiatry Neuropsychology and Behavioral Neurology. 1988;1:111-117
119. Barnes LL, Schneider JA, Boyle PA et al. Memory complaints are related to Alzheimer disease pathology in older persons. Neurology. 2006;67:1581-1585
120. Taylor HL, Jacobs DR, Jr., Schucker B et al. A questionnaire for the assessment of leisure time physical activities. J Chronic Dis. 1978;31:741-755
121. Morris JC. The Clinical Dementia Rating (CDR): current version and scoring rules. Neurology. 1993;43:2412b-2414
122. Hughes CP, Berg L, Danziger WL et al. A new clinical scale for the staging of dementia. British Journal of Psychiatry. 1982;140:566-572
123. Berg L, McKeel DW, Jr., Miller JP et al. Clinicopathologic Studies in Cognitively Healthy Aging and Alzheimer Disease: Relation of Histologic Markers to Dementia Severity, Age, Sex, and Apolipoprotein E Genotype. Archives of Neurology. 1998;55:326-335
124. Royall DR, Lauterbach EC, Kaufer D et al. The cognitive correlates of functional status: a review from the Committee on Research of the American Neuropsychiatric Association. J Neuropsychiatry Clin Neurosci. 2007;19:249-265

125. Bell-McGinty S, Podell K, Franzen M et al. Standard measures of executive function in predicting instrumental activities of daily living in older adults. Int J Geriatr Psychiatry. 2002;17:828-834
126. Cahn-Weiner DA, Boyle PA, Malloy PF. Tests of executive function predict instrumental activities of daily living in community-dwelling older individuals. Appl Neuropsychol. 2002;9:187-191
127. Jefferson AL, Paul RH, Ozonoff A, Cohen RA. Evaluating elements of executive functioning as predictors of instrumental activities of daily living (IADLs). Arch Clin Neuropsychol. 2006;21:311-320
128. Barry AJ, Steinmetz JR, Page HF, Rodahl K. The effects of physical conditioning on older individuals. II. Motor performance and cognitive function. J Gerontol. 1966;21:192-199
129. Powell RR. Psychological Effects of Exercise Therapy Upon Institutionalized Geriatric Mental-Patients. Journals of Gerontology. 1974;29:157-161
130. Gordon BA, Rykhlevskaia EI, Brumback CR et al. Neuroanatomical correlates of aging, cardiopulmonary fitness level, and education. Psychophysiology. 2008
131. Ismail AH, El-Naggar AM. Effect of exercise on cognitive processing in adult men. J Hum Ergol (Tokyo). 1981;10:83-91
132. Williams P, Lord SR. Effects of group exercise on cognitive functioning and mood in older women. Aust N Z J Public Health. 1997;21:45-52
133. Beekly DL, Ramos EM, Lee WW et al. The National Alzheimer's Coordinating Center (NACC) database: the Uniform Data Set. Alzheimer Dis Assoc Disord. 2007;21:249-258
134. Ball K, Berch DB, Helmers KF et al. Effects of cognitive training interventions with older adults: a randomized controlled trial. Jama. 2002;288:2271-2281
135. Grober E, Buschke H, Crystal H et al. Screening for dementia by memory testing. Neurology. 1988;38:900-903
136. Blumenthal JA, Emery CF, Madden DJ et al. Long-term effects of exercise on psychological functioning in older men and women. J Gerontol. 1991;46:P352-361
137. Prakash RS, Snook EM, Erickson KI et al. Cardiorespiratory fitness: A predictor of cortical plasticity in multiple sclerosis. Neuroimage. 2007;34:1238-1244
138. Wechsler D. Administration and scoring manual: Wechsler Memory Scale. San Antonio, TX: Harcourt Brace, 1997
139. Fabre C, Chamari K, Mucci P et al. Improvement of cognitive function by mental and/or individualized aerobic training in healthy elderly subjects. Int J Sports Med. 2002;23:415-421
140. Hill RD, Storandt M, Malley M. The impact of long-term exercise training on psychological function in older adults. J Gerontol. 1993;48:P12-17
141. Khatri P, Blumenthal JA, Babyak MA et al. Effects of exercise training an cognitive functioning among depressed older men and women. Journal of Aging and Physical Activity. 2001;9:43-57
142. Molloy DW, Beerschoten DA, Borrie MJ et al. Acute effects of exercise on neuropsychological function in elderly subjects. J Am Geriatr Soc. 1988;36:29-33
143. Molloy DW, Richardson LD, Crilly RG. The effects of a three-month exercise programme on neuropsychological function in elderly institutionalized women: a randomized controlled trial. Age Ageing. 1988;17:303-310
144. Shay KA, Roth DL. Association between aerobic fitness and visuospatial performance in healthy older adults. Psychol Aging. 1992;7:15-24
145. Goodglass H, Kaplan E. The assessment of aphasia and related disorders. Philadelphia: Lea & Febiger, 1983
146. El-Naggar AM. Physical training effect on relationship of physical, mental, and emotional fitness in adult men. J Hum Ergol (Tokyo). 1986;15:79-84
147. Blumenthal JA, Emery CF, Madden DJ et al. Cardiovascular and behavioral effects of aerobic exercise training in healthy older men and women. J Gerontol. 1989;44:M147-157
148. Madden DJ, Blumenthal JA. Interaction of hypertension and age in visual selective attention performance. Health Psychol. 1998;17:76-83
149. Hassmen P, Ceci R, Backman L. Exercise for older women: a training method and its influences on physical and cognitive performance. Eur J Appl Physiol Occup Physiol. 1992;64:460-466
150. Thurstone LL, Thurstone LG. Examiner Manual for the SRA Primary Mental Abilities Test. Chicago: Science Research Associates, 1949
151. Goodglass H, Kaplan E. Boston Diagnostic Apahsia Examination. Philadelphia: Lea & Febiger, 1983

152. Emery CF, Schein RL, Hauck ER, MacIntyre NR. Psychological and cognitive outcomes of a randomized trial of exercise among patients with chronic obstructive pulmonary disease. Health Psychol. 1998;17:232-240
153. Emery CF, Shermer RL, Hauck ER et al. Cognitive and psychological outcomes of exercise in a 1-year follow-up study of patients with chronic obstructive pulmonary disease. Health Psychol. 2003;22:598-604
154. Dustman RE, Emmerson RY, Ruhling RO et al. Age and fitness effects on EEG, ERPs, visual sensitivity, and cognition. Neurobiol Aging. 1990;11:193-200
155. Dustman RE, Ruhling RO, Russell EM et al. Aerobic exercise training and improved neuropsychological function of older individuals. Neurobiol Aging. 1984;5:35-42
156. Heaton RK, Chelune GJ, Talley JL et al. Wisconsin Card Sorting Test Manual: revised and expanded. Odessa, FL: Psychological Assessment Resources 1993
157. Armitage SG. An analysis of certain psychological tests used in the evaluation of brain injury. Psychological Monographs. 1946;60:1-48
158. Emery CF. Effects of age on physiological and psychological functioning among COPD patients in an exercise program. J Aging Health. 1994;6:3-16
159. Emery CF, Gatz M. Psychological and cognitive effects of an exercise program for community-residing older adults. Gerontologist. 1990;30:184-188
160. Hassmen P, Koivula N. Mood, physical working capacity and cognitive performance in the elderly as related to physical activity. Aging (Milano). 1997;9:136-142
161. Izquierdo-Porrera A, Waldstein SR. Cardiovascular Risk Factors and Cognitive Function in African Americans. The Journals of Gerontology. 2002;57B:P377
162. Wechsler D. Adminstration and scoring manual: Wechsler Adult Intelligence Scale. San Antonio, TX: Harcourt Brace, 1997
163. Hillman CH, Erickson KI, Kramer AF. Be smart, exercise your heart: exercise effects on brain and cognition. Nat Rev Neurosci. 2008;9:58-65
164. Blair SN, Cheng Y, Holder JS. Is physical activity or physical fitness more important in defining health benefits? Medicine And Science In Sports And Exercise. 2001;33:S379-S399
165. Hacker M, Stork S, Stratakis D et al. Relationship between right ventricular ejection fraction and maximum exercise oxygen consumption: a methodological study in chronic heart failure patients*1. Journal of Nuclear Cardiology. 2003;10:644-649
166. Fleg JL, Morrell, C. H., Bos, A. G., et al. Accelerated Longitudinal Decline of Aerobic Capacity in Healthy Older Adults. Circulation. 2005;112:674-682
167. Duncan GE, Howley ET, Johnson BN. Applicability of VO2max criteria: discontinuous versus continuous protocols. Medicine And Science In Sports And Exercise. 1997;29:273-278
168. Nagi SZ. An epidemiology of disability among adults in the United States. Milbank Mem Fund Q Health Soc. 1976;54:439-467
169. Shah KR, Carr D, Roe CM et al. Impaired Physical Performance and the Assessment of Dementia of the Alzheimer Type. Alzheimer Disease and Associated Disorders. 2004;18:112-118
170. Podsiadlo D, Richardson S. The timed "Up & Go": a test of basic functional mobility for frail elderly persons. J Am Geriatr Soc. 1991;39:142-148
171. Al Snih S, Markides KS, Ottenbacher KJ, Raji MA. Hand grip strength and incident ADL disability in elderly Mexican Americans over a seven-year period. Aging Clin Exp Res. 2004;16:481-486
172. Kohrt WM. Preliminary evidence that DEXA provides an accurate assessment of body composition. Journal of Applied Physiology. 1998;84:372-377
173. Pintauro SJ, Nagy TR, Duthie CM, Goran MI. Cross-calibration of fat and lean measurements by dual-energy X-ray absorptiometry to pig carcass analysis in the pediatric body weight range. American Journal of Clinical Nutrition. 1996;63:293-298
174. Collin C, Wade DT, Davies S, Horne V. The Barthel ADL Index: a reliability study. Int Disabil Stud. 1988;10:61-63
175. Mahoney FI, Barthel DW. Functional Evaluation: The Barthel Index. Md State Med J. 1965;14:61-65
176. Galasko D, Bennett D, Sano M et al. An inventory to assess activities of daily living for clinical trials in Alzheimer's disease. The Alzheimer's Disease Cooperative Study. Alzheimer Disease & Associated Disorders. Suppl 2:S33-9, 1997. 1997;11:S33-39
177. Stewart AL, Mills KM, Sepsis PG et al. Evaluation of CHAMPS, a physical activity promotion program for older adults. Ann Behav Med. 1997;19:353-361

178. Stewart AL, Verboncoeur CJ, McLellan BY et al. Physical Activity Outcomes of CHAMPS II: A Physical Activity Promotion Program for Older Adults. Journals of Gerontology Series A: Biological Sciences and Medical Sciences. 2001;56:M465-470
179. King AC, Pruitt LA, Phillips W et al. Comparative effects of two physical activity programs on measured and perceived physical functioning and other health-related quality of life outcomes in older adults. J Gerontol A Biol Sci Med Sci. 2000;55:M74-83
180. Ware JE, Jr., Sherbourne CD. The MOS 36-item short-form health survey (SF-36). I. Conceptual framework and item selection. Medical Care. 1992;30:473-483
181. Hamilton M. Development of a rating scale for primary depressive illness. Br.J Soc Clin Psychol. 1967;6:278-296
182. Hollenberg M, Haight T, Tager IB. Depression decreases cardiorespiratory fitness in older women. Journal of Clinical Epidemiology. 2003;56:1111-1117
183. Kaplan D. Structural Equation Modeling: Foundations and Extensions. Thousand Oaks, CA: Sage, 2000
184. Browne MW, R. RC. Alternative Ways of Assessing Model Fit. In: Bollen KA, Long JS, eds. Testing structural equation models. Newbury Park, CA: Sage, 1993:136-162
185. MacCallum R, Browne M. Testing differences between nested covariance structure models: Power analysis and null hypotheses. Psychological Methods. 2006;11:19-35
186. Johnson DK, Wilkins CH, Morris JC. Accelerated Weight Loss May Precede Diagnosis in Alzheimer Disease. Arch Neurol. 2006;63:1312-1317
187. Shrout PE, Bolger N. Mediation in experimental and nonexperimental studies: new procedures and recommendations. Psychological methods. 2002;7:422-445
188. Hanley JA, McNeil BJ. A method of comparing the areas under receiver operating characteristic curves derived from the same cases. Radiology. 1983;148:839-843
