# Supplementary material for: Dose-Response of Aerobic Exercise on Cognition: A Community-Based, Pilot Randomized Controlled Trial
Source: PLoS One. 2015 Jul 9;10(7):e0131647. doi: 10.1371/journal.pone.0131647 (PMC4497726; doi:10.1371/journal.pone.0131647)
Supplement: S1 Table — (DOCX) [file pone.0131647.s004.docx]

**S1 Table. Description and baseline performance on component cognitive tests.**

|  | **Control**  **(n=25)** | **75min/wk**  **(n=25)** | **150min/wk**  **(n=27)** | **225min/wk**  **(n=24)** |  |
| --- | --- | --- | --- | --- | --- |
| MMSE | 29.3 (0.9) | 29.2 (0.9) | 29.3 (1.2) | 29.2 (1.1) | Standard cognitive screener. Scored as 0-30 |
| Logical Memory (31) | 14.5 (3.4) | 14.6 (3.0) | 14.7 (3.2) | 15.7 (3.3) | Story read to participant. Scored as units remembered immediately and after >20 minutes. Scored 0-25. |
| Delayed Logical Memory (31) | 13.3 (3.4) | 13.4 (3.7) | 13.2 (3.6) | 13.8 (3.7) |  |
| Selective Reminding Task - Free Recall Total (32) | 27.8 (4.8) | 28.0 (4.7) | 27.7 (5.7) | 28.3 (5.1) | Sum of Free Recall. Scored 0-48. |
| Boston Naming Test (33) | 28.4 (1.5) | 28.1 (2.0) | 28.4 (1.5) | 28.4 (1.9) | Number of remembered, learned pictures. Scored 0-30 |
| Block Design (31) | 33.6 (11.9) | 34.9 (9.7) | 33.6 (11.8) | 33.6 (12.4) | Arrangement of blocks to mimic a presented patterns. Scored as seconds to complete design. |
| Stroop Color Reading (31) | 72.4 (10.7) | 74.1 (11.0) | 71.0 (13.2) | 74.5 (10.1) | Series of color of ink verbalized in 45 seconds. |
| Digit Symbol Substitution (31) | 46.7 (10.0) | 47.9 (8.0) | 47.3 (10.7) | 49.1 (9.6) | Series of symbols and corresponding numbers on paper. Scored as number of correct substitutions of symbols for numbers in 90 seconds, 0-93. |
| Trailmaking A (34) | 30.4 (8.1) | 28.0 (7.7) | 31.7 (11.5) | 28.1 (8.6) | Pattern of numbered circles on page to be connected in order. Scored as seconds to complete. |
| Digit Span-Forward (31) | 8.2 (1.5) | 8.8 (1.8) | 8.3 (1.9) | 8.5 (2.2) | Sequence of numbers read to participant. Scored as number of sequences repeated correctly, 0-12. |
| Digit Span-Backward (31) | 6.4 (2.0) | 7.1 (2.3) | 6.3 (2.3) | 6.4 (2.1) |  |
| Letter Number Sequencing (31) | 10.2 (2.6) | 10.4 (1.9) | 9.6 (2.1) | 10.0 (2.4) | Sequence of numbers and letters read to participant. Scored as number of sequences reordered correctly, 0-21. |
| DKEFS Card Sort-Free Sort Description (35) | 17.0 (4.8) | 17.3 (6.5) | 16.9 (6.7) | 17.3 (5.6) | Categorical sorting of cards. Scored as logical correct sorts. |
| DKEFS Card Sort-Confirmed Correct Sorts (35) | 2.9 (1.1) | 2.9 (1.4) | 2.9 (1.4) | 2.9 (1.1) |  |
| Verbal Fluency-Animals (33) | 19.6 (4.4) | 22.8 (5.9) | 21.9 (5.9) | 20.8 (5.8) | Number of unique items in a category that can be verbalized in 1 minute. Scored 0-77. |
| Verbal Fluency-Vegetables (33) | 15.6 (4.0) | 15.9 (4.6) | 14.8 (4.1) | 17.3 (5.1) |  |
| Inductive Reasoning-Letter (36) | 7.0 (2.7) | 7.7 (2.7) | 6.7 (3.4) | 7.7 (2.8) | Pattern completion. Scored as number correct, 0-15. |
| Inductive Reasoning–Word (36) | 8.0 (2.2) | 8.2 (2.3) | 7.0 (2.9) | 8.4 (2.3) |  |
| Matrix Reasoning (31) | 12.9 (4.2) | 12.0 (4.1) | 12.8 (5.8) | 12.0 (5.1) | Pattern completion. Scored as number correct, 0-26. |

Values are mean (standard deviation)
