# Supplementary material for: Dose-Response of Aerobic Exercise on Cognition: A Community-Based, Pilot Randomized Controlled Trial
Source: PLoS One. 2015 Jul 9;10(7):e0131647. doi: 10.1371/journal.pone.0131647 (PMC4497726; doi:10.1371/journal.pone.0131647)
Supplement: S2 Table — (DOCX) [file pone.0131647.s005.docx]

**S2 Table. Weekly progression of exercise duration in minutes.**

|  | Week | | | | | | | | | |  |
| --- | --- | --- | --- | --- | --- | --- | --- | --- | --- | --- | --- |
| **Group** | 1 | 2 | 3 | 4 | 5 | 6 | 7 | 8 | 9 | 10-26 | Sum |
| 75 | 60 | 75 | 75 | 75 | 75 | 75 | 75 | 75 | 75 | 75 | 1935 |
| 150 | 60 | 75 | 96 | 118 | 139 | 150 | 150 | 150 | 150 | 150 | 3638 |
| 225 | 60 | 75 | 96 | 118 | 139 | 161 | 182 | 204 | 225 | 225 | 5085 |
