# Supplementary material for: Dose-Response of Aerobic Exercise on Cognition: A Community-Based, Pilot Randomized Controlled Trial
Source: PLoS One. 2015 Jul 9;10(7):e0131647. doi: 10.1371/journal.pone.0131647 (PMC4497726; doi:10.1371/journal.pone.0131647)
Supplement: S3 Table — The table shows that the 5 derived cognitive domains were identically configured and loaded consistently onto the domain factors at baseline and 6-month follow-up. (DOCX) [file pone.0131647.s006.docx]

**S3 Table. Table of log-likelihood values for SEM-LRS models showing the 5 derived cognitive domains.**

|  | **Verbal Memory** | **GFI** | **CFI** | **Chi Square** | **d-2LL** | **DF** | **dChiDF** | **p Value** | **t(DF)** | **p Value** | **Model Accepted?** |
| --- | --- | --- | --- | --- | --- | --- | --- | --- | --- | --- | --- |
| 1 | Baseline Configuration | 1.00 | 1.00 |  |  |  |  |  |  |  |  |
| 2 | Longitudinal Configuration | 1.00 | 0.98 | 108.9 |  | 100 |  |  |  |  |  |
| 3 | Longitudinal Weak Invariance | 1.00 | 0.98 | 113.7 |  | 103 |  |  |  |  |  |
| 4 | Longitudinal Strong Invariance | 1.00 | 0.97 | 111.7 |  | 98 |  |  |  |  |  |
| 5 | Null Hypothesis |  |  | 148.7 |  | 111 |  |  |  |  |  |
| 6 | Practice Effect |  |  | ***118.9*** | -29.8 | 109 | -2 | p < .001 | 4.55 | p < 0.01 | ***Yes*** |
| 7 | Intervention Effect |  |  | 127.3 | -21.4 | 109 | -2 | p < .001 | 3.91 | p < 0.01 | No |
| 8 | Linear Dose Response |  |  | 127.3 | -21.4 | 109 | -2 | p < .001 | 3.91 | p < 0.01 | No |
| Ref | All parameters free to vary |  |  | 109.6 | -39.1 | 103 | -8 | p < .001 |  |  |  |
|  | **Visuospatial Processing** |  |  |  |  |  |  |  |  |  |  |
| 1 | Baseline Configuration | 1.00 | 0.95 |  |  |  |  |  |  |  |  |
| 2 | Longitudinal Configuration | 0.99 | 0.91 | 149.5 |  | 108 |  |  |  |  |  |
| 3 | Longitudinal Weak Invariance | 0.99 | 0.90 | 161.9 |  | 111 |  |  |  |  |  |
| 4 | Longitudinal Strong Invariance | 0.99 | 0.90 | 158.8 |  | 106 |  |  |  |  |  |
| 5 | Null Hypothesis |  |  | 190.8 |  | 119 |  |  |  |  |  |
| 6 | Practice Effect |  |  | 174.3 | -16.5 | 117 | -2 | p < .001 | 2.08 | p < 0.05 | No |
| 7 | Intervention Effect |  |  | 173.1 | -17.7 | 117 | -2 | p < .001 | 2.41 | p < 0.05 | No |
| 8 | Linear Dose Response |  |  | ***172.7*** | -18.0 | 116 | -3 | p < .001 | 1.99 | p < 0.05 | ***Yes*** |
| Ref | All parameters free to vary |  |  | 168.5 | -22.3 | 111 | -8 | p < .001 |  |  |  |
|  | **Attention** |  |  |  |  |  |  |  |  |  |  |
| 1 | Baseline Configuration | 1.00 | 0.98 |  |  |  |  |  |  |  |  |
| 2 | Longitudinal Configuration | 0.98 | 0.98 | 53.0 |  | 48 |  |  |  |  |  |
| 3 | Longitudinal Weak Invariance | 0.97 | 0.97 | 57.6 |  | 51 |  |  |  |  |  |
| 4 | Longitudinal Strong Invariance | 0.98 | 0.98 | 51.2 |  | 46 |  |  |  |  |  |
| 5 | Null Hypothesis |  |  | 66.6 |  | 57 |  |  |  |  |  |
| 6 | Practice Effect |  |  | 47.8 | -18.8 | 55 | -2 | p < .001 | 1.70 | ns | No |
| 7 | Intervention Effect |  |  | ***44.7*** | -22.0 | 55 | -2 | p < .001 | 2.42 | p < 0.05 | ***Yes*** |
| 8 | Linear Dose Response |  |  | 44.7 | -22.0 | 54 | -3 | p < .001 | 1.12 | ns | No |
| Ref | All parameters free to vary |  |  | 39.6 | -27.1 | 49 | -8 | p < .001 |  |  |  |
|  | **Set Maintenance and Shifting** |  |  |  |  |  |  |  |  |  |  |
| 1 | Baseline Configuration | 1.00 | 1.00 | 0.1 |  |  |  |  |  |  |  |
| 2 | Longitudinal Configuration | 0.97 | 1.00 | 56.7 |  | 56 |  |  |  |  |  |
| 3 | Longitudinal Weak Invariance | 0.97 | 1.00 | 59.7 |  | 59 |  |  |  |  |  |
| 4 | Longitudinal Strong Invariance | 0.97 | 1.00 | 56.0 |  | 54 |  |  |  |  |  |
| 5 | Null Hypothesis |  |  | 105.7 |  | 65 |  |  |  |  |  |
| 6 | Practice Effect |  |  | ***87.6*** | -18.1 | 63 | -2 | p < .001 | 1.26 | ns | ***No*** |
| 7 | Intervention Effect |  |  | 88.3 | -17.4 | 63 | -2 | p < .001 | 0.51 | ns | No |
| 8 | Linear Dose Response |  |  | 87.4 | -18.3 | 62 | -3 | p < .001 | 1.14 | ns | No |
| Ref | All parameters free to vary |  |  | 83.9 | -21.8 | 57 | -8 | p < .001 |  |  |  |
|  | **Reasoning** |  |  |  |  |  |  |  |  |  |  |
| 1 | Baseline Configuration | 1.00 | 0.99 |  |  |  |  |  |  |  |  |
| 2 | Longitudinal Configuration | 0.96 | 1.00 | 46.3 |  | 48 |  |  |  |  |  |
| 3 | Longitudinal Weak Invariance | 0.96 | 1.00 | 47.0 |  | 51 |  |  |  |  |  |
| 4a | Longitudinal Strong Invariance | 0.92 | 0.95 | 128.0 |  | 46 |  |  |  |  |  |
| 4b | Longitudinal Strong Invariance (Partial) | 0.96 | 1.00 | 43.0 |  | 42 |  |  |  |  |  |
| 5 | Null Hypothesis |  |  | 79.9 |  | 53 |  |  |  |  |  |
| 6 | Practice Effect |  |  | ***45.8*** | -34.1 | 51 | -2 | p < .001 | 2.43 | p < 0.05 | ***Yes*** |
| 7 | Intervention Effect |  |  | 48.6 | -31.3 | 51 | -2 | p < .001 | 1.73 | ns | No |
| 8 | Linear Dose Response |  |  | 48.5 | -31.3 | 50 | -3 | p < .001 | 1.43 | ns | No |
| Ref | All parameters free to vary |  |  | 43.9 | -35.9 | 45 | -8 | p < .001 |  |  |  |
