# Supplementary material for: Dose-Response of Aerobic Exercise on Cognition: A Community-Based, Pilot Randomized Controlled Trial
Source: PLoS One. 2015 Jul 9;10(7):e0131647. doi: 10.1371/journal.pone.0131647 (PMC4497726; doi:10.1371/journal.pone.0131647)
Supplement: S4 Table — Values are normalized mean and standard error (M = 0, SE = 1). Each value represents change in the group over 26 weeks. (DOCX) [file pone.0131647.s007.docx]

**S4 Table. Standardized change scores for the latent and component subtest scores in the Intent-to-Treat (n=101) and Per-Protocol Cohorts (n=77).**

|  | 26-Week Change | | | | | | | |
| --- | --- | --- | --- | --- | --- | --- | --- | --- |
| **Intent-to-Treat Cohort** | Control | | 75min/wk | | 150min/wk | | 225min/wk | |
|  | 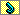Mean | (SE) | Mean | (SE) | 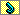Mean | (SE) | Mean | (SE) |
| **Verbal Memory Change** | | | | | | | | |
| Latent Residual Scores | 0.52 | (0.09) | 0.61 | (0.09) | 0.47 | (0.09) | 0.32 | (0.09) |
| *Logical Memory* | 0.30 | (0.18) | 0.55 | (0.18) | 0.19 | (0.15) | 0.22 | (0.22) |
| *Delayed Logical Memory* | 0.36 | (0.2) | 0.46 | (0.18) | 0.31 | (0.17) | 0.38 | (0.22) |
| *Selective Reminding Task - Free Recall Total* | 0.10 | (0.21) | 0.19 | (0.22) | 0.38 | (0.19) | 0.34 | (0.22) |
| *Boston Naming Test* | 0.36 | (0.14) | 0.23 | (0.17) | 0.42 | (0.11) | 0.11 | (0.2) |
| **Visuospatial Processing Change** | | | | | | | | |
| Latent Residual Scores | -0.02 | (0.03) | 0.19 | (0.03) | 0.08 | (0.03) | 0.29 | (0.03) |
| *Block Design* | 0.03 | (0.22) | 0.26 | (0.19) | -0.05 | (0.21) | 0.25 | (0.2) |
| *Stroop Color Reading* | -0.15 | (0.22) | 0.17 | (0.22) | -0.09 | (0.23) | 0.46 | (0.2) |
| *Digit Symbol Substitution* | -0.20 | (0.2) | 0.23 | (0.21) | 0.00 | (0.22) | 0.33 | (0.16) |
| *Trailmaking A* | -0.22 | (0.18) | 0.28 | (0.16) | 0.03 | (0.26) | 0.04 | (0.18) |
| **Simple Attention Change** | | | | | | | | |
| Latent Residual Scores | -0.06 | (0.09) | 0.11 | (0.09) | 0.09 | (0.09) | 0.14 | (0.09) |
| *Digit Span Forward* | -0.10 | (0.2) | 0.39 | (0.23) | -0.04 | (0.2) | 0.11 | (0.21) |
| *Digit Span Backward* | 0.00 | (0.18) | 0.26 | (0.22) | -0.11 | (0.19) | 0.17 | (0.23) |
| *Letter Number Sequencing* | -0.02 | (0.21) | 0.04 | (0.23) | -0.12 | (0.25) | -0.13 | (0.22) |
| **Set Maintenance & Shifting Change** | | | | | | | | |
| Latent Residual Scores | 0.07 | (0.32) | -0.08 | (0.32) | 0.14 | (0.32) | 0.05 | (0.32) |
| *DKEFS Card Sort -Free Sort Description* | 0.07 | (0.16) | 0.13 | (0.24) | 0.10 | (0.21) | -0.11 | (0.21) |
| *DKEFS Card Sort -Confirmed Correct Perceptual* | 0.02 | (0.14) | -0.03 | (0.2) | 0.12 | (0.2) | 0.01 | (0.2) |
| *Category Fluency*  *(Animal + Vegetable)* | -0.39 | (0.15) | 0.04 | (0.25) | -0.16 | (0.18) | 0.12 | (0.2) |
| **Reasoning Change** | | | | | | | | |
| Latent Residual Scores | 0.24 | (0.12) | 0.08 | (0.12) | 0.12 | (0.12) | 0.21 | (0.12) |
| *Inductive Reasoning Letter* | 0.66 | (0.21) | 0.61 | (0.21) | 0.47 | (0.25) | 0.89 | (0.21) |
| *Inductive Reasoning Word* | 0.18 | (0.21) | 0.27 | (0.23) | -0.06 | (0.25) | 0.43 | (0.2) |
| *Matrix Reasoning* | 0.14 | (0.22) | 0.03 | (0.16) | -0.17 | (0.18) | -0.05 | (0.18) |
|  | | | | | | | | |
| **Per-Protocol Cohort** | Control | | 75min/wk | | 150min/wk | | 225min/wk | |
|  | 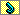Mean | (SE) | Mean | (SE) | 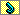Mean | (SE) | Mean | (SE) |
| **Verbal Memory Change** | | | | | | | | |
| Latent Residual Scores | 0.51 | (0.17) | 0.69 | (0.18) | 0.39 | (0.15) | 0.45 | (0.19) |
| *Logical Memory* | 0.41 | (0.18) | 0.73 | (0.21) | 0.16 | (0.17) | 0.37 | (0.29) |
| *Delayed Logical Memory* | 0.43 | (0.19) | 0.63 | (0.19) | 0.25 | (0.18) | 0.48 | (0.28) |
| *Selective Reminding Task - Free Recall Total* | 0.15 | (0.2) | 0.30 | (0.24) | 0.32 | (0.21) | 0.32 | (0.27) |
| *Boston Naming Test* | 0.37 | (0.14) | 0.33 | (0.15) | 0.37 | (0.12) | 0.09 | (0.26) |
| **Visuospatial Processing Change** | | | | | | | | |
| Latent Residual Scores | -0.03 | (0.11) | 0.21 | (0.11) | 0.12 | (0.13) | 0.25 | (0.11) |
| *Block Design* | 0.00 | (0.24) | 0.21 | (0.2) | -0.03 | (0.24) | 0.34 | (0.27) |
| *Stroop Color Reading* | -0.16 | (0.21) | 0.16 | (0.25) | -0.02 | (0.26) | 0.57 | (0.23) |
| *Digit Symbol Substitution* | -0.10 | (0.21) | 0.35 | (0.25) | -0.08 | (0.26) | 0.56 | (0.18) |
| *Trailmaking A* | -0.26 | (0.19) | 0.29 | (0.16) | 0.16 | (0.27) | 0.17 | (0.23) |
| **Simple Attention Change** | | | | | | | | |
| Latent Residual Scores | -0.05 | (0.13) | 0.17 | (0.2) | 0.26 | (0.13) | 0.17 | (0.17) |
| *Digit Span Forward* | -0.12 | (0.22) | 0.31 | (0.27) | 0.03 | (0.23) | 0.19 | (0.31) |
| *Digit Span Backward* | 0.09 | (0.18) | 0.35 | (0.26) | 0.00 | (0.22) | 0.42 | (0.29) |
| *Letter Number Sequencing* | 0.00 | (0.2) | 0.14 | (0.25) | 0.18 | (0.25) | -0.06 | (0.29) |
| **Set Maintenance & Shifting Change** | | | | | | | | |
| Latent Residual Scores | 0.09 | (0.12) | -0.09 | (0.13) | 0.11 | (0.13) | 0.04 | (0.15) |
| *DKEFS Card Sort -Free Sort Description* | 0.10 | (0.17) | 0.23 | (0.28) | 0.10 | (0.26) | 0.09 | (0.24) |
| *DKEFS Card Sort -Confirmed Correct Perceptual* | 0.07 | (0.14) | -0.01 | (0.23) | 0.07 | (0.22) | 0.16 | (0.26) |
| *Category Fluency*  *(Animal + Vegetable)* | -0.24 | (0.16) | 0.30 | (0.3) | -0.11 | (0.21) | 0.08 | (0.27) |
| **Reasoning Change** | | | | | | | | |
| Latent Residual Scores | 0.27 | (0.15) | 0.34 | (0.17) | 0.12 | (0.17) | 0.26 | (0.21) |
| *Inductive Reasoning Letter* | 0.66 | (0.2) | 0.77 | (0.21) | 0.21 | (0.24) | 1.02 | (0.27) |
| *Inductive Reasoning Word* | 0.25 | (0.21) | 0.59 | (0.23) | -0.21 | (0.24) | 0.61 | (0.28) |
| *Matrix Reasoning* | 0.24 | (0.22) | 0.25 | (0.17) | -0.20 | (0.2) | -0.05 | (0.25) |

Values are normalized mean and standard error (M=0, SE=1). Each value represents change in the group over 26 weeks.
