# Supplementary material for: Dose-Response of Aerobic Exercise on Cognition: A Community-Based, Pilot Randomized Controlled Trial
Source: PLoS One. 2015 Jul 9;10(7):e0131647. doi: 10.1371/journal.pone.0131647 (PMC4497726; doi:10.1371/journal.pone.0131647)
Supplement: S5 Table — Data represent mean change (SE) in cognitive domain latent factors over 26 weeks (unconstrained latent mean change scores [M = 0, SE = 1]). Hypothesis Testing: If differences were present at the group level in 26-week cognitive outcomes, we used a nested contrast analysis to characterize whether those differences fit one of three patterns: (1) Practice Effect, equivalent improvement across all groups; (2) Intervention Effect, equivalent improvement across exercise doses; (3) Linear Dose-Response, linear improvement across exercise doses. The most parsimonious models accepted by the nested comparison procedures are reported in bold. Degrees of Freedom (df) for SEM models are the difference between the number of parameters estimated in unrestricted structural model and the number of parameters estimated in hypothesis driven (constrained) model. ∅ indicates that the trend model did not fit. * 26-week change scores for Set Maintenance & Shifting were different than zero. However, nested contrast testing failed to reach significance indicating no distinct pattern of change from baseline. (DOCX) [file pone.0131647.s008.docx]

**S5 Table. Group latent factor score estimates, best fitting trend models, and change in cognitive domain subtest Z-scores (M=0, SE=1) in the Per-Protocol Cohort.**

|  | **26-Week Change** | | | | | | | | | | | **Hypothesis Testing** |
| --- | --- | --- | --- | --- | --- | --- | --- | --- | --- | --- | --- | --- |
| **Per-Protocol Cohort**  **(n=77)** | Control  (n=23) | | 75min/wk  (n=18) | | | 150min/wk  (n=21) | | | 225min/wk  (n=15) | | |  |
|  | 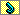Mean | (SE) | Mean | | (SE) | 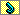Mean | | (SE) | Mean | (SE) | |  |
| **Verbal Memory Change** | | | | | | | | | | | | |
| Latent Residual Scores | 0.51 | (0.17) | 0.69 | | (0.18) | 0.39 | (0.15) | | 0.45 | | (0.19) | Omnibus Test  ΔX^2^ = 39.1 (Δdf=8),  p < 0.001 |
| - **Practice Effect** | **0.52** | **(0.11)** | **0.52** | | **(0.11)** | **0.52** | **(0.11)** | | **0.52** | | **(0.11)** | **ΔX^2^ = 29.8 (Δdf=1)**  **model** **accepted** |
| - Exercise Effect* | 0.39 | (0.12) | 0.47 | | (0.12) | 0.47 | (0.12) | | 0.47 | | (0.12) | Δ*X*^2^ = 21.4 (Δ*df*=2)  model rejected |
| - Dose Response* | 0.39 | (0.12) | 0.47 | | (0.12) | 0.47 | (0.12) | | 0.47 | | (0.12) | Δ*X*^2^ = 21.4 (Δ*df*=3)  model rejected |
| **Visuospatial Processing Change** | | | | | | | | | | | | |
| Latent Residual Scores | -0.03 | (0.11) | 0.21 | (0.11) | | 0.12 | (0.13) | | 0.25 | | (0.11) | Omnibus Test  ΔX^2^ = 22.3 (Δdf=8),  p < 0.001 |
| - Practice Effect | 0.13 | (0.07) | 0.13 | (0.07) | | 0.13 | (0.07) | | 0.13 | | (0.07) | Δ*X*^2^ = 16.5 (Δ*df*=1)  model rejected |
| - Exercise Effect | -0.03 | (0.10) | 0.18 | (0.07) | | 0.18 | (0.07) | | 0.18 | | (0.07) | Δ*X*^2^ = 17.7 (Δ*df*=2)  model rejected |
| - **Dose Response** | **-0.02** | **(0.10)** | **0.13** | **(0.08)** | | **0.13** | **(0.08)** | | **0.24** | | **(0.12)** | **ΔX^2^ = 18.0 (Δdf=3)**  **model** **accepted** |
| **Simple Attention Change** | | | | | | | | | | | | |
| Latent Residual Scores | -0.05 | (0.13) | 0.17 | (0.20) | | 0.26 | (0.13) | | 0.17 | | (0.17) | Omnibus Test  ΔX^2^ = 27.1 (Δdf =8),  p < 0.001 |
| - Practice Effect | -- | -- | -- | -- | | -- | -- | | -- | | -- | ∅ |
| - **Exercise Effect** | **-0.07** | **(0.13)** | **0.23** | **(0.09)** | | **0.23** | **(0.09)** | | **0.23** | | **(0.09)** | **ΔX^2^ = 22.0 (Δdf=2)**  **model accepted** |
| - Dose Response | -- | -- | -- | -- | | -- | -- | | -- | | -- | ∅ |
| **Set Maintenance & Shifting Change** | | | | | | | | | | | | |
| Latent Residual Scores | 0.09 | (0.12) | -0.09 | (0.13) | | 0.11 | (0.13) | | 0.04 | | (0.15) | Omnibus Test  ΔX^2^ = 21.8 (Δdf =8),  p < 0.001* |
| Practice Effect | -- | -- | -- | -- | | -- | -- | | -- | | -- | ∅ |
| Exercise Effect | -- | -- | -- | -- | | -- | -- | | -- | | -- | ∅ |
| Dose Response | -- | -- | -- | -- | | -- | -- | | -- | | -- | ∅ |
| **Reasoning Change** | | | | | | | | | | | | |
| Latent Residual Scores | 0.27 | (0.15) | 0.34 | (0.17) | | 0.12 | (0.17) | | 0.26 | | (0.21) | Omnibus Test  ΔX^2^ = 35.9 (Δdf =8),  p < .001 |
| - **Practice Effect** | **0.23** | **(0.10)** | **0.23** | **(0.10)** | | **0.23** | **(0.10)** | | **0.23** | | **(0.10)** | **ΔX^2^ = 34.1 (Δdf=1)**  **model accepted** |
| - Exercise Effect | -- | -- | -- | -- | | -- | -- | | -- | | -- | ∅ |
| - Dose Response | -- | -- | -- | -- | | -- | -- | | -- | | -- | ∅ |

Data represent mean change (SE) in cognitive domain latent factors over 26 weeks (unconstrained latent mean change scores [M=0, SE=1]). *Hypothesis Testing:* If differences were present at the group level in 26-week cognitive outcomes, we used a nested contrast analysis to characterize whether those differences fit one of three patterns: (1) Practice Effect, equivalent improvement across all groups; (2) Intervention Effect, equivalent improvement across exercise doses; (3) Linear Dose-Response, linear improvement across exercise doses. The most parsimonious models accepted by the nested comparison procedures are reported in bold. Degrees of Freedom (df) for SEM models are the difference between the number of parameters estimated in unrestricted structural model and the number of parameters estimated in hypothesis driven (constrained) model. ∅ indicates that the trend model did not fit. * 26-week change scores for Set Maintenance & Shifting were different than zero. However, nested contrast testing failed to reach significance indicating no distinct pattern of change from baseline.
